# Sodium Chloride Cotransporter in Hypertension

**DOI:** 10.3390/biomedicines12112580

**Published:** 2024-11-11

**Authors:** Annalisa Castagna, Gabriele Mango, Nicola Martinelli, Luigi Marzano, Sara Moruzzi, Simonetta Friso, Francesca Pizzolo

**Affiliations:** 1 Department of Medicine, University of Verona, 37134 Verona, Italy; annalisa.castagna@univr.it (A.C.); gabriele.mango@univr.it (G.M.); nicola.martinelli@univr.it (N.M.); simonetta.friso@univr.it (S.F.); 2 Unit of Internal Medicine B, Department of Medicine, University of Verona School of Medicine, Azienda Ospedaliera Universitaria Integrata Verona, Policlinico “G.B. Rossi”, 37134 Verona, Italy; luigi.marzano@aovr.veneto.it (L.M.); sara.moruzzi@aovr.veneto.it (S.M.)

**Keywords:** sodium chloride cotransporter (NCC), hypertension, urinary extracellular vesicles

## Abstract

The sodium chloride cotransporter (NCC) is essential for electrolyte balance, blood pressure regulation, and pathophysiology of hypertension as it mediates the reabsorption of ultrafiltered sodium in the renal distal convoluted tubule. Given its pivotal role in the maintenance of extracellular fluid volume, the NCC is regulated by a complex network of cellular pathways, which eventually results in either its phosphorylation, enhancing sodium and chloride ion absorption from urines, or dephosphorylation and ubiquitination, which conversely decrease NCC activity. Several factors could influence NCC function, including genetic alterations, hormonal stimuli, and pharmacological treatments. The NCC’s central role is also highlighted by several abnormalities resulting from genetic mutations in its gene and consequently in its structure, leading to dysregulation of blood pressure control. In the last decade, among other improvements, the acquisition of knowledge on the NCC and other renal ion channels has been favored by studies on extracellular vesicles (EVs). Dietary sodium and potassium intake are also implicated in the tuning of NCC activity. In this narrative review, we present the main cornerstones and recent evidence related to NCC control, focusing on the context of blood pressure pathophysiology, and promising new therapeutical approaches.

## 1. Introduction

**Figure 1:**
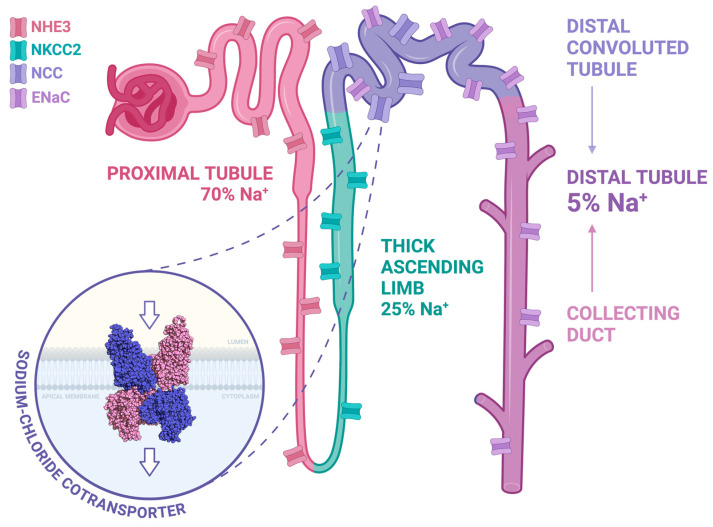
Sodium reabsorption along the nephron. Most sodium reabsorption occurs in the proximal tubule (pink) via NHE3, which mediates about 70% of the Na^+^ transport, followed by NKCC2 in the thick ascending limb (green), which is responsible for 25% of transport. About 5% of Na^+^ reabsorption occurs in the distal tubule, which includes the distal convoluted tubule (purple) and the collecting duct (magenta). The NCC is located only in the proximal part of the distal convoluted tubule, while ENaC is present in both the distal convoluted tubule and the collecting duct. A representation of the NCC dimeric structure is also illustrated in the image. Created in BioRender. Friso, S. (2026) https://BioRender.com/7ozy4sj (accessed on 8 November 2024).

Arterial hypertension is one of the most common chronic diseases, affecting an estimated 1.3 billion individuals worldwide. It is one of the world’s leading risk factors for death and disability, with a still low rate of blood pressure (BP) control despite the availability of several efficacious anti-hypertensive drugs [1]. Arterial hypertension is secondary to identifiable underlying conditions only in a minority of cases; in most cases, its cause is unidentified, and in such a case, it is called primary. Primary hypertension is considered a multifactorial disease, with numerous factors interacting to raise blood pressure and determine end-organ damage. Several different mechanisms cooperate in the pathophysiology of arterial hypertension, among which are genetic predisposition, volume expansion related to increased water and salt absorption at the kidney level, impaired modulation of the renin–angiotensin–aldosterone system (RAAS), and increased stimulation of the sympathetic nervous system (SNS). All of these features may lead to either the development of increased total peripheral resistance or an increased afterload, influencing the development of hypertension [2]. The kidney is the central actor in the control of water–sodium balance [3,4]. Sodium homeostasis in the kidney is modulated by the activation of tubular ion channels, mediating the reabsorption of more than 99% filtered sodium (Figure 1). Progressively from proximal to distal renal tubules, the main channels are (i) the sodium–hydrogen exchanger isoform 3 (NHE3); (ii) the Na^+^-K^+^-2Cl^−^ cotransporter (NKCC2); (iii) the sodium chloride cotransporter (NCC); and (iv) the amiloride-sensitive epithelial sodium channel (ENaC). The first two mediate the greatest amount of sodium absorption, with about 70% of it obtained by NHE3 in the proximal tubules and about 25% by NKCC2 in the thick ascending limb of the Henle loop. The remaining 5% of sodium absorption in the distal tubule is mainly modulated by aldosterone by means of ENaC activation in the distal tubule and collecting duct and NCC regulation at the distal convoluted tubule (DCT). Of note, ENaC activation is modulated not only by aldosterone but also by other hormones non-necessarily binding to the mineralocorticoid receptor (MR), such as vasopressin [5].

NCC-mediated ion transport in humans has recently gained growing attention due to the emerging evidence of its interaction with relatively novel diuretic (and not only) drugs, as well as the well-known targeted therapy with thiazides. Moreover, the NCC’s central role is underlined by the detection of several genetic variants and, consequently, abnormalities in its structure and function, eventually leading to the dysregulation of blood pressure. In the last decade, the acquisition of a deeper knowledge on the NCC and other renal ion channels was favored by studies in extracellular vesicles (EVs) such as exosomes [6]. Urinary extracellular vesicles (UEVs)—and, in particular, exosomes secreted by renal tubular epithelial cells and easily detectable in urine—can carry proteins, nucleic acids, and lipids, mirroring the composition of tubular cells; therefore, they are particularly useful as a liquid biopsy for studying kidney proteins/mechanisms [7,8].

In this narrative review, we specifically focus on the NCC channel for its recognized emerging role in blood pressure control, highlighting the state of the art on the pathophysiology of its modulation and its role as a target for pharmacological therapies.

## 2. NCC Structure and Function

The NCC, encoded by the *SLC12A3* gene, is a member of the electroneutral cation–chloride cotransporter (CCC) family that mediates Na^+^ and Cl^−^ cotransport following a 1:1 stoichiometry [9,10]. It shares approximately 50% sequence identity with the other two Na^+^-coupled Cl^−^ cotransporters belonging to this group, namely, NKCC1 and NKCC2 [11]. The NCC is the most important sodium transporter in the apical membrane of the renal DCT, where, together with ENaC, it is responsible for the reabsorption of the ultrafiltered Na^+^, playing an important role in the regulation of extracellular fluid (ECF) volume, potassium homeostasis, urine dilution, and blood pressure [12].

The reabsorption of NaCl load in the DCT, causing the consequent variation of Na^+^ and Cl^−^ intracellular gradient, affects, in turn, the homeostasis of other ions, such as K^+^, Ca^2+^, and Mg^2+^, by modulating their reabsorption in the distal nephron [12,13].

In the distal DCT, the reabsorption of Ca^2+^ and Mg^2+^ varies inversely and directly, respectively, with the NCC cotransport activity in the proximal DCT. Multiple lines of evidence indicate that NCC inhibition may contribute to hypomagnesemia by reducing the drive for apical Mg^2+^ entry through the inhibition of Na^+^-K^+^-ATPase [14,15]. Magnesium deficiency itself may promote Mg^2+^ loss by directly decreasing Na^+^-K^+^-ATPase activity, as magnesium acts as a cofactor, and reduced intracellular Mg^2+^ levels may lead to the further downregulation of the NCC, worsening the condition. In contrast, inappropriate activation of the NCC does not appear to affect Mg^2+^ handling [15]. On the other hand, Ca^2+^ reabsorption is related to the calcium sensing receptor (CaSR), which, in turn, is recognized to activate the NCC [16]. Although NCC does not act on K^+^ reabsorption itself (not being able to transport K^+^), it strongly influences the transport of this ion by affecting the amount of Na^+^ in cells expressing ENaC in distal DCT and CNT. When NCC activity increases, a lower amount of Na^+^ is available in the distal tubules, with a parallel reduction in K^+^ secretion in the urine. By contrast, when NCC activity decreases, the increase in distal Na^+^ to be reabsorbed leads to a higher K^+^ excretion.

**Figure 2:**
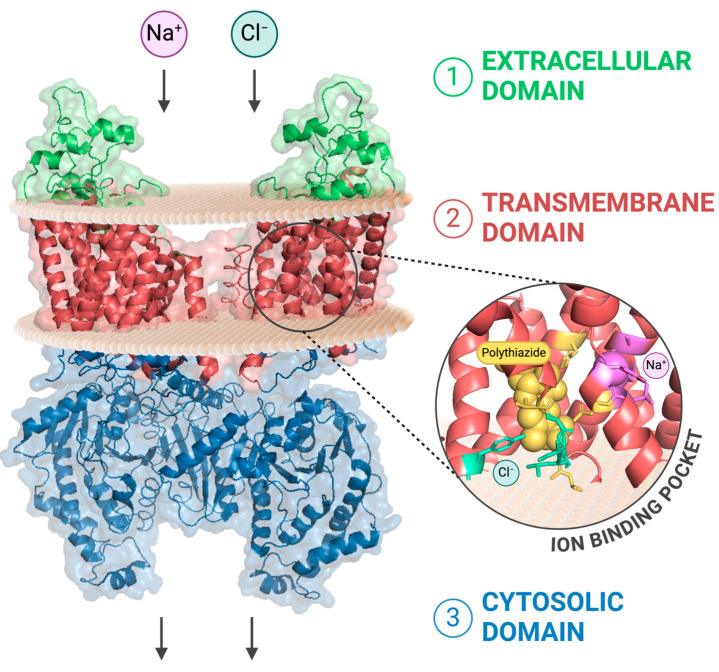
Three-dimensional structure of the NCC dimer embedded in the apical cell membrane. This schematic representation highlights the functional domains of the transporter: (1) the extracellular domain (green); (2) the transmembrane domain (red); and (3) the cytosolic domain (blue). A close-up of one ion-binding pocket, located within the transmembrane domain, shows three binding sites: one for Na^+^ (magenta); one for Cl^−^ (cyan); and one for polythiazide, a thiazide diuretic (yellow). The representation is based on the structural prediction provided by the Orientations of Proteins in Membranes (OPM) database [21] of the NCC inward-facing conformation obtained by Fan et al. [19] (PDB ID: 8FHO). PyMOL (Schrödinger, LLC) was used to visualize the structure and generate the image. Created in BioRender. Friso, S. (2026) https://BioRender.com/qdks1mm (accessed on 8 November 2024).

From a structural point of view, the NCC is a homodimer formed by two subunits assembled with a domain-swap organization [17]. Each subunit is composed of: (a) a transmembrane domain (TMD), which is primarily involved in ion binding and transport, and consists of 12 transmembrane helices adopting a LeuT-fold conformation; (b) an extracellular domain, that comprehends two ordered loops acting as a gate controlling the access to the ion binding pocket present in the TMD; (c) two cytoplasmic regulatory domains, composed of the N- and C-terminal ends respectively (Figure 2) [18,19]. Each monomer forms a solvent-accessible vestibule, harboring four ion binding sites, two for Na^+^ and two for Cl^−^. To allow for ion translocation, the NCC undergoes a conformational switch, alternatively exposing the ion binding sites localized in the central space of the vestibule to either the extracellular or the intracellular environment. In the outward-facing conformation, both Na^+^ and Cl^−^ binding sites are accessible from the extracellular solvent; therefore, the ions can accommodate the binding pocket. On the other hand, the intracellular side of the vestibule is closed by transmembrane helixes which are tightly packed together, with a short helix blocking the substrate translocation pathway [19]. After ion binding, the carrier rearranges in an inward-facing conformation, revealing a cytosolic exit pathway for ions delimited by polar residues. Conversely, the extracellular gate is closed thanks to a conserved salt bridge in the CCC family [20], with a hydrophobic constriction formed by nonpolar residues that prevents ions from escaping back into the extracellular space [18].

## 3. NCC Regulation

**Figure 3:**
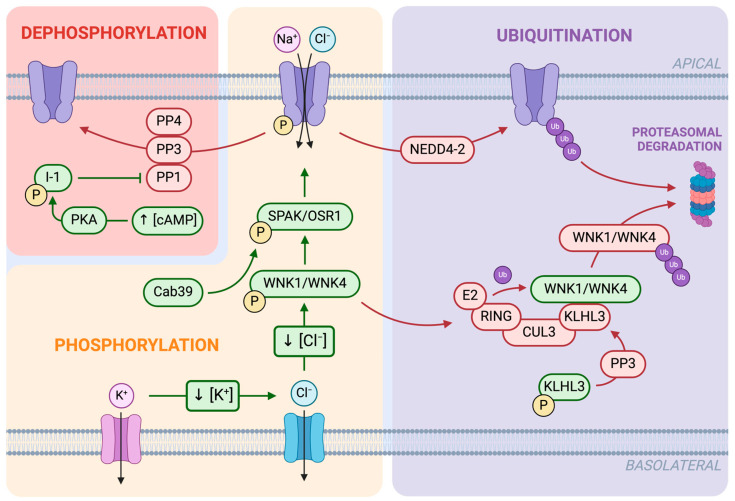
Post-translational modifications regulating NCC activity in the DCT. NCC phosphorylation (yellow) is primarily mediated by the WNK-SPAK/OSR1 kinase cascade. WNK1 and WNK4 (with-no-lysine kinases) activate SPAK (STE20/SPS1-related proline/alanine-rich kinases) and OSR1 (oxidative stress-response protein 1), which, in turn, phosphorylate the NCC, increasing its transport activity in the apical membrane. WNK autophosphorylation is stimulated by low Cl^−^ intracellular levels, which result from a decrease in K^+^ intracellular concentration. These changes are, respectively, mediated by basolateral chlorine (light blue) and potassium channels (pink). Cab39 (calcium-binding protein 39) is another regulator of NCC phosphorylation since it is required for SPAK activation. NCC dephosphorylation (red) leads to a reduction in its activity and is carried out by protein phosphatases PP1, PP3, and PP4. PP1 is regulated by protein phosphatase 1 inhibitor-1 (I-1) through the cAMP/PKA pathway. A rise in cAMP levels activates PKA (protein kinase A), which phosphorylates I-1, preventing PP1 from dephosphorylating the NCC. Ubiquitination (purple) is another key regulatory mechanism that affects NCC function. WNK abundance is controlled by an E3 ubiquitin ligase complex composed of KLHL3 (Kelch-like protein 3) and CUL3 (Cullin 3). This complex tags the NCC with ubiquitin (Ub), marking it for proteasomal degradation. KLHL3 acts as an adapter for WNK, and its phosphorylation prevents this binding. PP3 is responsible for KLHL3 activation through dephosphorylation. Additionally, NEDD4-2, another ubiquitin ligase, directly mediates NCC degradation through ubiquitination. Created in BioRender. Friso, S. (2026) https://BioRender.com/ypw9014 (accessed on 8 November 2024).

The NCC is regulated by a complex network of cellular pathways, which eventually result in either its phosphorylation, dephosphorylation, or ubiquitination (Figure 3) [13].

Among these post-translational modifications, phosphorylation plays a pivotal role in the modulation of NCC function. NCC-mediated ion transport is activated through the phosphorylation of conserved threonine and serine residues in the cytosolic N-terminal domain of the transporter. The main phosphorylation sites of the human NCC are Thr46, Thr55, Thr60, Ser73, and Ser91 [22,23], which correspond to Thr53, Thr58, and Ser71 in mice [24]. Within these residues, Thr60 (Thr58 in mice) is critically important for NCC function, and mutating this single amino acid markedly inhibits NCC activity [25]. The addition of phosphate groups to these sites may induce conformational changes in the cytosolic regulatory domains that facilitate ion transport [19] or increase NCC abundance on the apical membrane [26,27]. NCC phosphorylation results from a kinase cascade stimulated by several signaling mediators, and the central node of each pathway is the activation of two members of the with-no-lysine (WNK) kinase family: WNK1 and WNK4 [28]. These enzymes do not directly interact with the NCC, but they phosphorylate and activate two other kinases, namely, STE20/SPS1-related proline/alanine-rich kinase (SPAK) and oxidative stress-response protein 1 (OSR1). SPAK and, to a lesser extent, OSR1 are the main kinases responsible for the phosphorylation of the NCC [29]. Several factors can influence the WNK-SPAK/OSR1-NCC cascade, including, for instance, the presence of calcium binding scaffolding proteins (Cab39 and Cab39l) [24], variations of Cl^−^, K^+^, and other ions concentrations [13], and various hormonal stimuli [30]. Calcium extracellular concentrations, for instance, have been demonstrated to regulate the NCC through the action of CaSR, which activates WNK4 [31].

Another post-translational modification that significantly alters NCC function is ubiquitination, which can modulate NCC activity either directly or indirectly. Direct NCC ubiquitination is mainly carried out by the ubiquitin–protein ligase NEDD4-2, and it results in NCC endocytosis and consequential degradation [32,33]. On the other hand, ubiquitination can also occur on WNK kinases involved in the upstream regulation of the NCC. This mechanism is mediated by an E3 ligase complex formed by Kelch-like protein 3 (KLHL3) and Cullin 3 (CUL3), directing WNK kinases to the proteasome and thus reducing NCC activity by downregulating its phosphorylation [34].

Lastly, the NCC’s sodium uptake can be inhibited through dephosphorylation, which could enhance the transporter internalization and ubiquitination [26]. Two phosphatases have been proven to directly catalyze the dephosphorylation of the NCC, namely, PP1 [35] and PP4 [36], while another enzyme, known as calcineurin or PP3, can decrease NCC phosphorylation both directly and indirectly. Calcineurin is not only able to mediate the dephosphorylation of the NCC [37] but is also responsible for the dephosphorylation of KLHL3, which promotes WNK4 ubiquitination and degradation, ultimately leading to lower phosphorylated NCC (pNCC) levels [38]. In addition, it is worth mentioning that PP1 activity is influenced by cAMP intracellular levels, which can stimulate the phosphorylation of I-1, an endogenous regulator of PP1. For this reason, an increase in cAMP concentration causes an inhibition of PP1, resulting in an increase in pNCC [39].

Various stimuli were demonstrated to modulate NCC activity. Early studies on animals and cells had indicated that the NCC is activated by mineralocorticoids, glucocorticoids, vasopressin, insulin, furosemide, a low potassium diet, sodium restriction, and female sex hormones, while a high K^+^ load, thiazides, and male sex hormones inhibit it [30,40,41,42,43].

**Figure 4:**
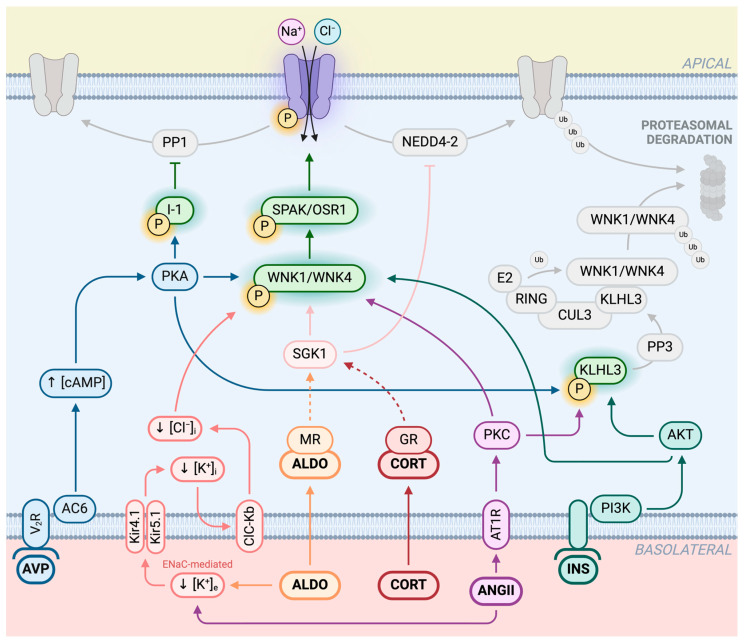
Physiological stimuli that modulate NCC activity. The key signaling pathways involved in NCC regulation by various hormones are illustrated. Arginine vasopressin (AVP, blue) binds to the V_2_ receptor (V_2_R) on the basolateral membrane, activating adenylate cyclase 6 (AC6), which increases cyclic AMP (cAMP) levels. Elevated cAMP stimulates protein kinase A (PKA), leading to (a) stimulation of WNK-SPAK/OSR1-NCC cascade through WNK kinase phosphorylation; (b) inhibition of protein phosphatase 1 (PP1) by phosphorylating its inhibitor, I-1, maintaining the phosphorylated state of the NCC; and (c) phosphorylation and inactivation of Kelch-like protein 3 (KLHL3), diminishing WNK ubiquitination and consequent degradation. Aldosterone (ALDO, orange) enhances the NCC in both a mineralocorticoid receptor (MR)-dependent and MR-independent manner. Binding to MR, it can stimulate serum- and glucocorticoid-regulated kinase 1 (SGK1), which activates WNKs and simultaneously inhibits NEDD4-2-mediated ubiquitination. On the other hand, aldosterone stimulates ENaC-mediated potassium secretion, eventually inducing hypokalemia. Low extracellular K^+^ levels are sensed by DCT cells through Kir4.1/Kir5.1 K^+^ channels, which, once active, lead to a drop in K^+^ intracellular concentration, stimulating basolateral Cl^-^ transport through ClC-Kb. A decrease in intracellular Cl^−^ levels releases the inhibition of WNK kinases, enabling their autophosphorylation and, thus, stimulating the WNK-SPAK/OSR1-NCC pathway. Cortisol (CORT, red) may exert the same effect as aldosterone, likely through the interaction with the glucocorticoid receptor (GR). Angiotensin II (ANGII, purple) binds to the angiotensin II type 1 receptor (AT1R), activating protein kinase C (PKC), which catalyzes both WNK and KLHL3 phosphorylation. Angiotensin II activates the NCC via AngII receptor type 1 (AT1R), stimulating the WNK4-SPAK kinase cascade and the phosphorylation of KLHL. Angiotensin II can also induce aldosterone and ENaC-mediated hypokalemia, thereby triggering potassium sensing through Kir4.1/Kir5.1 K^+^ channels. Insulin (INS, green) activates the phosphoinositide 3-kinase (PI3K) pathway, leading to the activation of protein kinase B (AKT). AKT stimulates NCC activity through the phosphorylation of both WNK kinases and KLHL3. Created in BioRender. Friso, S. (2026) https://BioRender.com/xp6rgf6 (accessed on 8 November 2024).

Here below, we illustrate some of the main mechanisms involved in NCC regulation by physiological stimuli (see Figure 4).

### 3.1. Insulin

Insulin receptors are ubiquitously expressed along the nephron, and it has been demonstrated that insulin increases sodium reabsorption by promoting the activity of the major renal sodium transporters [44].

In vitro and in vivo studies revealed that insulin induces NCC activation through the PI3K-AKT pathway, which can then stimulate WNK-SPAK/OSR1 cascade [45,46,47,48]. Once activated by insulin, AKT could phosphorylate KLHL3, thus reducing WNK4 degradation and increasing NCC phosphorylation [49].

### 3.2. Vasopressin

Arginine vasopressin (AVP), also called anti-diuretic hormone (ADH), plays a central role in water and sodium homeostasis in the kidney, increasing water reabsorption through acquaporin-2 in the distal nephron, but also mediating Na^+^ reabsorption in multiple sections of the nephron, including DCT [50]. It has been shown that AVP increases NCC abundance and phosphorylation in Brattleboro rats through the stimulation of WNK-SPAK/OSR cascade [51]. AVP effect is mediated through binding to the vasopressin V_2_ receptor (V_2_R), which activates adenylate cyclase 6, promoting cAMP production [52]. A rise in cAMP intracellular levels activates protein kinase A (PKA), which could enhance NCC phosphorylation acting on multiple targets. PKA can directly phosphorylate WNK4 [53,54] or modulate its abundance through the phosphorylation of KLHL3, that inhibits WNK4 degradation [49]. AVP can also mediate the phosphorylation of I-1, reducing NCC dephosphorylation through the inhibition of PP1 [54]. Other mediators such as CAMKK and PI3K have been proposed as putative regulators of AVP-induced NCC activation, with PKA possibly playing a minor role [55].

### 3.3. Angiotensin II

There is compelling evidence that angiotensin II (AngII) activates the NCC via AngII receptor type 1 (AT1R), stimulating the WNK4-SPAK kinase cascade independently of aldosterone in vitro [56,57,58,59]. The signaling cascade is mediated by protein kinase C (PKC), which modulates WNK4 activity through its direct phosphorylation [53] and through the phosphorylation of KLHL3, thus preventing WNK4 degradation and increasing its abundance [60]. However, animal studies showed that K^+^ supplementation can override the stimulatory effect of AngII on the NCC, suggesting that AngII-induced NCC activation is likely secondary to hypokalemia mediated by ENaC stimulation [61].

### 3.4. Glucocorticoids

Animal studies revealed that NCC phosphorylation levels have diurnal fluctuations influenced by glucocorticoids through a signaling pathway that is likely mediated by the glucocorticoid receptor (GR) [62]. Adult inducible nephron-specific GR knockout mice exhibited lower NCC activation [63], while glucocorticoid excess induced non dipping blood pressure, which was restored to normal with thiazide diuretics administration [64]. The precise molecular mechanisms triggered by glucocorticoids in DCT remain to be elucidated, even though several mediators have been proposed to influence the WNK-SPAK/OSR1-NCC cascade, including serum and glucocorticoid regulated kinase 1 (SGK1) [65], glucocorticoid-induced leucine zipper protein (GILZ) [66], and the circadian clock protein Per1 [67].

### 3.5. Sex Hormones

A sexual dimorphism in NCC activity has been observed in both animal models and in human-derived UEVs, with females presenting higher levels of total and phosphorylated NCC, possibly due to the action of estrogens, progesterone, and prolactin [68,69]. Female rodents displayed a higher cellular density of the NCC in the DCT compared to male subjects [70,71], and this differential expression is partially controlled by estrogens, since ovariectomized female rats showed similar NCC levels compared to males, and a higher NCC abundance was, in part, restored through estradiol administration [41]. This sexual dimorphism could carry relevant clinical implications, particularly in guiding the choice of the strategy for managing hypertension. Studies on hypertensive postmenopausal women receiving hydrochlorothiazide, an NCC inhibitor, revealed that hormone replacement therapy could enhance the BP-lowering effect of this drug, also mitigating its adverse hemodynamic and metabolic effects [72,73].

### 3.6. Aldosterone

Aldosterone increases NCC activation with mechanisms not only mediated by its binding to MR but also by potassium sensing.

It is well known that aldosterone increases NCC activation, and for decades, in fact, there was a general agreement on the direct role of aldosterone on regulating the NCC, supported by studies showing an increase in the pNCC after aldosterone infusion [74,75]. However, more recent evidence supports also an indirect role of aldosterone on NCC regulation [76]. NCC activation by aldosterone can, in fact, be secondary to eventual changes in K^+^ plasma concentration. MRs can modulate NCC activation, acting through plasma K^+^ levels and ENaC. When aldosterone is in excess, ENaC is directly stimulated, leading to a decrease in K^+^ extracellular concentration. Low K^+^ levels secondarily act on NCC activation, further enhancing salt retention and hypertension. Aldosterone is synthesized and then passively released into the bloodstream in response to two primary triggers: high potassium levels and low blood volume. The glomerulosa cells have a high potassium conductance due to the presence of G protein-activated inwardly rectifying potassium channels (GIRK4) on their cell membranes. As a result, an increase in plasma potassium concentration depolarizes these cells, which, in turn, activates voltage-dependent calcium channels. This leads to a rise in intracellular calcium levels, which stimulates the synthesis of aldosterone. On the other hand, hypovolemia leads to the activation of the RAAS, which, in turn, stimulates aldosterone synthesis by the activation of AT1R. The aldosterone’s action depends on its interaction with the MR. In the principal cells of the connecting tubule (CNT) and cortical collecting ducts, the activated MR prompts the transcription of several genes, leading ultimately to increased activity of the ENaC. ENaC, through its activity of sodium reabsorption, generates a lumen-negative transepithelial voltage that promotes potassium excretion through the outer medullary potassium channel (ROMK), also present in the apical membrane of these cells. These processes primarily enhance renal potassium excretion in response to hyperkalemia and sodium retention in response to hypovolemia. The matter on the direct or indirect action of aldosterone anyway remains controversial [76,77], and other pathways could also be involved, such as activation of EGFR-dependent signaling [78], a direct action on the WNK-OSR1/SPAK-NCC cascade [79], and the modulation of NCC protein expression via a pathway involving SGK1 and NEDD4-2 [80].

### 3.7. Potassium

The extracellular potassium concentration significantly influences NCC phosphorylation [13,81]. DCT cells detect changes in K^+^ levels through the basolateral potassium channel Kir4.1/Kir5.1. Low extracellular K^+^ causes membrane hyperpolarization and reduction in intracellular Cl^−^ concentration, dismissing the inhibition of WNK4 phosphorylation and allowing the WNK-SPAK pathway to phosphorylate and activate the NCC. The increase in NaCl reabsorption induces a reduction in distal tubular Na delivery and limitation of kaliuresis. Conversely, high extracellular K^+^ levels dephosphorylate the NCC, decreasing NaCl reabsorption and promoting potassium excretion (kaliuresis). There is increasing evidence that a high K diet influences NCC activation also by additional mechanisms [82]. The effects of high K to dephosphorylate the NCC occur rapidly (within minutes), and the speed of the response has led to the hypothesis that phosphatases are involved in NCC dephosphorylation, probably mediated by a change in intracellular Ca^2+^ levels. The increase in intracellular Ca^2+^ concentration can influence the calcium-binding protein calmodulin and, subsequently, protein phosphatases such as calcineurin. This causes dephosphorylation of the NCC, thereby reducing NaCl reabsorption through the cotransporter. The effect of a low potassium concentration on the NCC phosphorylation is reported in Figure 3.

## 4. Alteration of NCC Activity

Given the key role of the NCC in maintaining ion balance, pH, and extracellular fluid volume, NCC-related disorders may result in clinically relevant systemic effects. These alterations can be directly caused by defects of the NCC itself or dysfunction occurring in either signaling pathways or different ion transport systems that ultimately affect NCC-mediated Na^+^-Cl^−^ transport [83].

### 4.1. Decrease in NCC Activity

#### 4.1.1. Gitelman Syndrome

Gitelman syndrome is the most common form of hereditary tubulopathy, with a prevalence of 1–10/40,000 [84]. It is an autosomal recessive disease characterized by the presence of loss-of-function variants on *SLC12A3* gene. A lower NCC activity results in a reduction of the extracellular fluid volume driven by an increase in Na^+^, K^+^, Mg^2+^, and H^+^ renal wasting, which causes hypokalemia, metabolic alkalosis, hypomagnesemia, hypocalciuria, and hyperreninemic hyperaldosteronism with associated hypotension [85]. Mouse models with deletion of the NCC were studied and presented similar phenotypes as Gitelman syndrome, with mild dysregulations of fluid volume homeostasis and sodium [86].

#### 4.1.2. Bartter Syndrome

A lower NCC activity can also be caused by an increase in Cl^−^ concentrations within DTC cells due to defects in the outward transport through the basolateral membrane. High Cl^−^ intracellular levels may result from an autosomal recessive renal salt wasting disorder called Bartter syndrome (BS). BS is classified in five subtypes according to the molecular mechanisms that are affected (BSI, II, III, IVa, IVb, V). The NCC function could be altered by BS type III and IV, which are characterized by loss of function variants occurring at the level of *CLCKA* and *CLCKB* (or both as for BSIVb), encoding for voltage-gated Cl^−^ channels, and *BSND*, encoding for a protein required for the insertion of these channels into the plasma membrane [87]. BS type V, differently to all the other BS types which are inherited in an autosomal recessive manner, is X-linked recessive, transient antenatal, and caused by mutations in the gene encoding the protein melanoma-associated antigen D2 (MAGE-D2) [88]. If Cl^−^ efflux is impaired, Cl^−^ accumulates inside DTC cells, diminishing the electrochemical gradient and contemporary downregulating WNK pathway, thereby reducing NCC activity [89,90] (Figure 3).

#### 4.1.3. Other Genetic Defects

A rise in K^+^ intracellular concentration due to defects in the outward K^+^ transport can similarly affect NCC function by increasing intracellular Cl^−^ concentration, suppressing WNK-SPAK/OSR1-NCC cascade and subsequently NCC activation [91] (Figure 3). Loss of function variants occurring on *KCNJ16* and *KCNJ10*, which encode for two basolateral K^+^ channel subunits, Kir4.1 and Kir5.1, create a condition with a clinical presentation that resembles NCC-related tubulopathy, in the context of a more complex syndrome affecting also the central nervous system [92]. Moreover, a decrease in Na^+^-K^+^ ATPase pump activity can also lead to an increase in K^+^ and Cl^−^ intracellular levels, which eventually inhibit NCC function [83]. Ultimately, a reduction in NCC activity could be caused by alterations of a wide range of molecular mechanisms, which often result in a phenotype very similar and comparable to that of Gitelman syndrome. Therefore, genetic testing is recommended, when possible, for better disease characterization and a proper differential diagnosis [93].

### 4.2. Increase in NCC Activity

#### Gordon Syndrome

Differently from those inducing a loss of function, *SLC12A3* variants that generate a gain of function are yet unknown, while it is well established that NCC overactivation can be indirectly achieved by monogenic alterations affecting genes involved in regulating NCC activity. These alterations are due to certain genetic variants that fall under the umbrella of Gordon syndrome, pseudo hypoaldosteronism type 2,or familial hyperkalemic hypertension (FHHt), a Mendelian form of hypertension. The prevalence of this rare disease is unknown, and it can be inherited as either autosomal dominant or recessive [94]. Phenotypically, Gordon syndrome is the mirror of Gitelman syndrome, being characterized by an enhanced Na^+^ and Cl^−^ reabsorption in the distal nephron, which leads to hyperkalemia, metabolic acidosis and hypertension [95]. This condition is caused by an overactivation of the WNK-SPAK/OSR1-NCC pathway, either through gain of function of WNK1 and WNK4 or loss of function of KLHL3 and CUL3 [96]. In either case, an increased abundance of WNK kinases due to a lower ubiquitination rate occurs, resulting in higher NCC phosphorylation and activity. The WNK-SPAK/OSR1-NCC cascade has been actively studied for identifying novel therapeutic targets to use in FHHt for reducing blood pressure [97]. Mouse models overexpressing WNK1 present increased phosphorylation of the NCC [98], whereas knockout mouse models of WNK4 presented lower levels of phosphorylated NCC and NKCC2 [99]. NCC activity is highly regulated by a complex signaling network, and these kinases appear to be sensitive to changes in hormonal and physiological environments.

## 5. NCC and Hypertension

The NCC is an important target of the main mechanisms involved in hypertension pathophysiology—mainly sodium imbalance, but also increased SNS activity and changes in the RAAS regulation [100]. Blood pressure homeostasis is critically controlled by the renal system through the maintenance of electrolyte content and concentrations in the ECF, and Na^+^ reabsorption dysregulation is thus the main cause of abnormal water loss or retention [86]. Blood pressure dysregulation and water–sodium imbalances are caused by NCC protein dysfunctions. The increased activation of the NCC causes increased Na^+^ reabsorption, with the consequence of raising blood pressure, as observed with the genetically determined increase in NCC activation in Gordon syndrome. By contrast, loss of function variants in the *SLC12A3* gene in Gitelman syndrome are characterized by low blood pressure. In hypertensive disease, alongside the genetic causes of an altered NCC activity, special attention is devoted to potassium. Paradigmatically, the role of the NCC in the physiopathology of hypertension is also expressed in the context of K^+^ sensing by renal epithelial cells, called the “potassium switch” [101]. The switch suggests that low K^+^ intake acts as a trigger, linking K^+^ levels to NCC regulation. When dietary K^+^ intake is low, the switch “activates” the NCC, and when K^+^ intake is high, it “deactivates” the NCC. This adjustment influences the amount of sodium (Na^+^) delivered to downstream ENaC, thereby modulating Na^+^/K^+^ exchange and ECF K^+^ concentrations [102]. Thus, while aldosterone stimulates ENaC to enhance distal Na^+^ reabsorption and K^+^ excretion, ECF K^+^ concentration directly regulates NCC levels and phosphorylation [74,75,76]. In hyperaldosteronism, where a pathological aldosterone excess is present, both effects of its action (potassium excretion and hypovolemia) tend to occur simultaneously, leading to a form of hypertension with hypokalemia. Chronic effects of aldosterone excess on the NCC appear to be rather dependent on alterations in K^+^ balance. Kidney-specific MR knockout mice exhibited lower NCC and pNCC levels [103], but the effects of MR on the NCC are not so prominent compared to changes in K^+^ plasma concentration [76], and they are dispensable for NCC activation in mice [104]. Some authors suggest that hypokalemia is the major driver of NCC regulation [76,77,78,79].

### 5.1. Dietary Salt Influence on NCC

Salt intake in the diet is an important variable to consider as Na^+^ concentration influences aldosterone production and transporters activity at kidney levels. Aldosterone secretion is suppressed by a high Na^+^ sodium diet, inducing the elimination of sodium excess, while its synthesis is promoted by low Na^+^ diet intake with subsequent Na^+^ retention and volume maintenance. In the situation of primary aldosteronism (PA), affecting the 5–15% of hypertensive population, aldosterone levels are elevated regardless of K^+^ plasma concentration or ECF volume and not restored by high Na^+^ levels. Hyperaldosteronism is associated with a higher risk of major cardiovascular events and a worse outcome in hypertensive patients [105]. Nowadays, in Western societies, as well as in China and Japan, most people, including those with hypertension, chronically consume more than the recommended daily amount of Na^+^ [106,107]. Moreover, hyperaldosteronism raises the NaCl taste recognition threshold [108], causing patients affected by this form of hypertension to be at risk of high Na^+^ intake. Thus, in the contest of a pathologic dysregulation of aldosterone production, Na^+^ dietary intake effects assume a peculiar role. Some recent studies investigated the effect of aldosterone and high Na^+^ diet on DCT in terms of transporters abundance and activity and K^+^-mediated remodeling and on patients with PA’ clinical parameters [109,110]. Despite the lack of guidelines on Na^+^ intake for patients with PA, the beneficial role of a low sodium diet on BP and coronary artery disease is widely recognized. The work by Zhou and colleagues demonstrated that reducing dietary Na was effective in lowering BP and raising K^+^ serum concentration in patients with idiopathic hyperaldosteronism. Moreover, females were more likely to achieve BP control when on a low-sodium diet. The authors thus recommended a daily sodium intake of 50 mmol for those patients [110]. Unfortunately, the authors did not investigate the behavior of the NCC or other transporters, but it is plausible to assume that the modification of K^+^ blood concentration would affect the Na^+^ reabsorption at the DCT level. On this basis, an interesting work by Mutchler and colleagues recently showed, in a mouse model treated with high Na^+^ diet combined with chronic aldosterone treatment, that NCC and pNCC expression significantly increased with aldosterone, together with decreased blood K^+^ concentration. The addition of a high-salt diet greatly decreased the aldosterone-dependent NCC increment, despite equal levels of hypokalemia. The authors showed the behavior of ENaC as well, indicating an increase in α-subunit expression with aldosterone treatment regardless of Na^+^ [109]. The salt sensitivity of blood pressure has been observed in about 50% of hypertensive subjects and 25% of normotensives and can be triggered by dietary sodium intake in prone individuals [111]. Salt sensitive hypertension has been studied also in animal models (e.g., DAHL salt sensitive rat (DSS) model), clearly showing a sex dependent-effect of salt intake [112]. This latter aspect was recently illustrated by Kim and colleagues in DSS and Dahl salt resistant (DSR) rats [113], showing sex- and strain-dependent differential NCC regulation via the canonical WNK/SPAK/OSR1 pathway. They showed that in salt resistance and in both sexes, the downregulation of the NCC in response to high dietary salt intake is conserved, but only in male and not in female DSS rats does this activation contribute to the salt sensitivity. Interestingly, male rats exhibited decreased WNK4 expression and decreased SPAK and OSR1, while female rats only suppressed SPAK and OSR1. These data suggest a likely sex dependent impact of WNK signaling in NCC regulation. Female rats exhibited higher levels of NCC protein and activity in all the strains (DSR, DSS, and basal) and a lower magnitude of hypertension in DSS rats in comparison to male rats.

### 5.2. Sympathetic Regulation of NCC

Increasing interest has been shown in delineating the interactions between SNS and the kidney, which functions to regulate sodium reabsorption. SNS is indeed important in the pathogenesis of salt-sensitive hypertension since its increased activity can, in fact, trigger an increment in renal sodium and water retention [114]. Efferent renal sympathetic nerve activity (ERSNA) is inappropriately increased in the presence of sodium retention in pathophysiological sodium-retaining states, including hypertension. A shift from inhibitory to excitatory renorenal reflexes, likely due to activation of renal chemosensitive nerves, can be observed in conditions of renal disease [115]. In normal physiological states, inhibitory renorenal reflexes help maintain low ERSNA; an increase in ERSNA leads to an increase in afferent renal nerve activity (ARNA), which causes a reduction, with a feedback mechanism, of ERSNA, to prevent excessive sodium retention. However, in states of dysfunction, these inhibitory reflexes may be impaired, potentially leading to predominance of excitatory reflexes, contributing to hypertension and fluid retention. Sodium intake also plays a role as a high-sodium diet can enhance the responsiveness of renal sensory nerves, making them more sensitive to changes in sodium levels, while a low-sodium diet may dampen this sensitivity, allowing the body to adjust appropriately to different sodium intakes. A direct role of SNS release of norepinephrine (NE) on NCC expression and activity has been suggested, but studies on the impact of elevated NE levels on NCC expression during normal dietary salt intake have produced conflicting evidence. Increased plasma NE content, obtained by a subcutaneous infusion, in rodents, at normal salt intake, increased the renal NCC expression in some experiments but not in others [116,117,118]. Moreover, increased dietary salt intake suppresses sympathetic outflow and circulating NE levels in salt-resistant Sprague-Dawley rats [119] and downregulates NCC expression and activity [120]. The latter studies highlighted the importance of the interaction between NE and salt to modulate NCC function and expression in the pathophysiology of salt-sensitive hypertension, as well as the importance of using multiple animal models to investigate the pathways underlying salt sensitivity. Furthermore, a study by Frame and colleagues [121] highlighted that in salt-sensitive hypertension in male Sprague-Dawley rats, sympathetic excitation increases NCC activity via a NE-activated adrenoceptor-mediated signal transduction pathway that increases the expression and/or activity of NCC regulatory kinases WNK4 and SPAK/OSR1. The same group also showed that α_1_-adrenoceptor antagonism attenuated the development and maintenance of SSH in male DSS rats, in part, by suppressing NCC activity and regulation. Conversely, β-adrenoceptor antagonism failed to influence SSH, NCC activity, or regulation in male DSS rats [122]; however, the exact mechanism triggered by NE in DCT cells is still controversial.

Moreover, in the frame of an action of the sympathetic tone on blood pressure, aging was also demonstrated to play an important role. Evidence supports an age-related decrease in markers of the presence of the ARN in aged Sprague–Dawley rats [123], and recently, Frame et al. confirmed the hypothesis that an age-related impairment in the ARN sympatho-inhibitory renorenal reflex contributes to increased sympathetic flux and subsequent NCC-mediated renal sodium retention activated by NE. The aging-related development of hypertension and salt-sensitive BP was evident in male but not in female Sprague-Dawley rats [124].

## 6. NCC Role in Other Forms of Hypertension

### 6.1. Cushing Syndrome

Cushing syndrome is a condition characterized by an excess of glucocorticoids, especially cortisol. A frequent manifestation of cortisol excess is hypertension, characterizing about 75–80% of patients with Cushing syndrome [125,126]. The mechanism of glucocorticoid-induced hypertension is multifactorial and is still not fully understood; however, renal water and sodium retention play a relevant roles [125], with the possible involvement of NCC activation. In the kidneys, excess glucocorticoids are responsible for a global incrementation in renal blood flow and sodium reabsorption due to activation of both mineralocorticoid and glucocorticoid receptors [127]. In the rat kidney, the synthetic glucocorticoid dexamethasone was demonstrated to upregulate the apical Na^+^ transporters; THE ENaC, NHE3, NKCC2, and NCC [128,129]. Patients with Cushing syndrome and suppressed RAAS are characterized by lower potassium and higher phosphorylated NKCC2 and NCC in urinary extracellular vesicles compared to healthy subjects. Moreover, the increases in both pNKCC2 and pNCC are reversed by the treatment [130].

### 6.2. Eclampsia

Pre-eclampsia is a pregnancy complication characterized by hypertension, proteinuria, and renal water and sodium retention. The pathogenesis of pre-eclampsia is complex and includes endothelial injury, reduced nitric oxide synthesis, and glomerular dysfunction. Pre-eclamptic women display an avid sodium retention by the kidney, despite an inhibited RAAS [131,132]. To date, only few studies are available regarding the involvement of tubular ion channels in pre-eclampsia. To study the sodium transporters possibly responsible for sodium retention, the expression, proteolytic cleavage, and phosphorylation of sodium channels were examined in UEVs of eclamptic women compared to normal pregnant women and healthy non-pregnant controls. The differences observed were in line with an increased activity of NKCC2 and ENaC but with a reduction in the activation of the NCC. From this human study, therefore, the NCC does not seem to be one of the ion channels involved in sodium retention in pre-eclampsia [133]. More recently, in an animal model (lipopolysaccharide (LPS)-induced pre-eclampsia (PE) rats), the LPS-PE rats exhibited, in kidneys and urine exosomes, higher renal total and phosphorylated NKCC2 and NCC with elevated mRNAs and lower ubiquitinated NCC than controls [134]. Even if a misregulation in ion transport is probably involved in the pathogenesis of pre-eclampsia, further studies are necessary to elucidate the detailed role of renal sodium transporters in this disease.

## 7. Pharmacological Regulation of NCC

**Figure 5:**
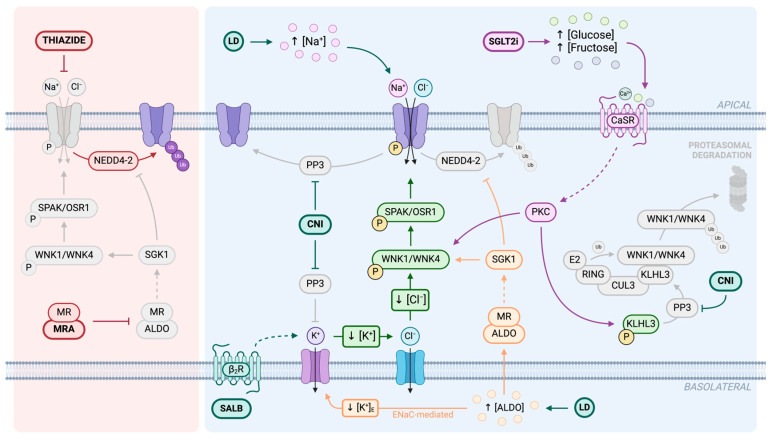
Pharmacological modulation of NCC function in the DCT. Inhibitors are shown in red, while enhancers are shown in light blue. Thiazide diuretics directly inhibit NCC, blocking ion translocation, while mineralocorticoid receptor antagonists (MRA) act indirectly, blocking the MR-mediated effect of aldosterone. Loop diuretics (LD) activate the NCC, increasing the amount of sodium delivered to the DCT and the secretion of aldosterone, which increases NCC activity in both an MR-dependent and an MR-independent manner. Calcineurin inhibitors (CNIs) decrease PP3-mediated dephosphorylation of NCC, basolateral K^+^ channel, and KLHL3, thus stimulating NCC activity at various levels. Salbutamol (SALB) could activate the basolateral K^+^ channel through a signaling pathway likely mediated by β_2_-adrenergic receptor. A drop in K^+^ intracellular concentration eventually triggers the WNK-SPAK/OSR1-NCC cascade. SGLT2 inhibitors (SGLT2i, shown in purple) may influence NCC activity, increasing the delivery of glucose and fructose to the DCT and possibly stimulating a signaling pathway involving the calcium-sensing receptor (CaSR) and protein kinase C (PKC). Created in BioRender. Friso, S. (2026) https://BioRender.com/yew9781 (accessed on 8 November 2024).

The NCC is one of the main targets for hypertension management given its central role in the regulation of blood pressure through electrolyte balance. It is directly inhibited by a broad class of drugs called “thiazide diuretics”, which prevent ion translocation by occluding its ion binding pocket, but there are also several other molecules which can alter NCC function indirectly, influencing its phosphorylation and ubiquitination state [100]. In recent years, novel inhibitors have been developed to modulate WNK-SPAK/OSR1-NCC pathway at various levels, but further testing is still required to reach the clinical trial phase [135]. We describe here below the principal market-approved drugs which are known to have an impact on NCC activity, either blocking or enhancing it (Figure 5).

### 7.1. NCC Activity Blockers

#### 7.1.1. Thiazide Diuretics

The term thiazide indicates a heterogenous class of diuretics that have been among the first-line medications for the treatment of hypertension since 1950s [2,136]. They are divided in two main groups according to their chemical structure: thiazide-type drugs, like hydrochlorothiazide, which contain a benzothiadiazine ring; and thiazide-like drugs, such as chlorthalidone and indapamide, which conversely lack of this ring. Within the class of thiazide diuretics, there is great variability in terms of both pharmacokinetic and pharmacodynamic properties, resulting in different dosing and frequency of administration [137]. In general, thiazide diuretics are among the most potent antihypertensive drugs, with chlorthalidone and indapamide displaying higher potency and a longer-lasting effect compared to hydrochlorothiazide, but a greater incidence of metabolic side effects of chlortalidone has been reported in some studies [138]. Even though they are primarily prescribed for their diuretic effect, certain thiazides have also pleiotropic properties that contribute to improve cardiovascular outcomes, reducing the risk of stroke and heart failure. However, it should be also noted that some thiazide diuretics have been associated with several metabolic complications, like hyperuricemia, glucose intolerance, and lipid abnormalities [139]. Hydrochlorothiazide was also correlated with an increased risk of non-melanoma skin cancer and melanoma in the general population [140]. Such correlation is related to ultraviolet light exposure that activates hydrochlorothiazide compounds and leads to the generation of reactive oxygen species, thereby triggering inflammation, cytotoxicity, and DNA damage [141]. Recent cryo-EM studies have revealed that thiazide diuretics act as orthosteric antagonists, competing with Na^+^ and Cl^−^ ions for the same binding sites. They inhibit NCC settling into a pocket approximately halfway of the ion translocation path, occluding the extracellular vestibule and blocking the transporter in the outward-open conformation [19,23].

#### 7.1.2. MR Antagonists

As it happens for loop diuretics (see paragraph below), it is reasonable to hypothesize an increase in NCC activity via sodium–water deprivation with all diuretic drugs, including MR antagonists (MRA). However, the modulation of the NCC by aldosterone is at least partially MR-mediated, with an expected reduction in NCC activation with drugs antagonizing MR. In animal studies, spironolactone demonstrated a negative modulation on the NCC. In rats with a high NCC renal abundance induced by treatment with loop diuretics, spironolactone decreased NCC protein abundance by 66%, compared with the furosemide-treated group [142]. The administration of spironolactone to rats after salt restriction decreased the abundances of both NCC and ENaC subunits in renal tubules [143]. Investigating the NCC phosphorylation modulated by OSR1/SPAK signaling, the increased aldosterone-mediated phosphorylation was reversed by treatment with spironolactone [79]. In the UEVs of hypertensive patients, our research group demonstrated that treatment with the MRA spironolactone was associated with increased NCC mRNA, with an inverse correlation between the basal renin concentration and NCC mRNA abundance after therapy. In other words, the MRA-treated patients with lower basal values of renin (i.e., more severe volume expansion) were also those with a greater change in the expression of NCC mRNA, mirroring a greater NCC response to diuretic therapy [144]. Today, alongside spironolactone, the first and widely used nonspecific MRA, other MRAs more specific to the aldosterone receptor are commercially available, such as eplerenone and finerenone, and others are promising in pre-commerce studies [145]. However, no data are available at present concerning their effect on the NCC except for eplerenone in mice, with an effect considered comparable to spironolactone [146,147].

### 7.2. NCC Activity Enhancers

#### 7.2.1. Loop Diuretics

Loop diuretics, by blocking sodium reabsorption in the thick ascending limb of the loop of Henle, increase the amount of sodium delivered to the DCT, provoking a tubular compensatory sodium reabsorption in DCT [148]. In healthy volunteers, chronic treatment with loop diuretics amplified ion transport rates in the distal tubule, estimated as the dose of thiazide diuretic able to inhibit the Na^+^ and Cl^−^ reabsorption [149]. The compensatory sodium reabsorption at nephron segments distal to the loop of Henle is one of the mechanisms responsible for the resistance to loop diuretics in heart failure that can be overcome by the consensual administration of a thiazide diuretic [150]. A direct influence of furosemide, a loop diuretic, on the NCC was demonstrated in animal studies [151]. Chronic loop diuretic administration increases the number of thiazide-sensitive NCCs [152], and the increased NCC protein abundance is partially mediated by aldosterone [142,153].

#### 7.2.2. Calcineurin Inhibitors

Calcineurin inhibitors are immunosuppressive drugs used to block T-cell activation after solid organ transplantation and in the treatment of various autoimmune disorders [154]. The two most prescribed drugs belonging to this group are cyclosporine and tacrolimus, which can bind to specific cytoplasmic receptors known as immunophilins. Once formed, the drug-receptor complex can competitively inhibit calcineurin activation [155]. Calcineurin inhibitor usage is associated with a series of side effects, including nephrotoxicity, hyperkaliemia, and hypertension [156]. The development or worsening of hypertension during treatment is very frequent, involving up to 30–60% of treated patients. The pathophysiology of calcineurin inhibitors induced hypertension is multifactorial; [157] furthermore, there is a compelling body of evidence indicating that chronic hypertension induced by calcineurin inhibitors is also mediated by NCC activation [158,159]. Calcineurin inhibitors can indeed enhance NCC activity acting at different levels: they prevent calcineurin-mediated NCC dephosphorylation as the result of a calmodulin-dependent pathway [160]; they inhibit KHE3 dephosphorylation, downregulating WNK ubiquitination and thereby increasing pNCC levels [38]; and they stimulate Kir4.1-Kir5.1 activation, enhancing the basolateral K^+^ efflux and leading to a drop in Cl^−^ intracellular concentration, which eventually triggers WNK-SPAK/OSR1-NCC cascade [161].

The hypothesis that NCC activation is a mechanism of CNI-induced hypertension could have a therapeutic consequence: CNI induced hypertension could be especially sensitive to thiazide diuretics, and seminal animal and human studies suggest this opportunity, even if studies clearly demonstrating the superiority of thiazides over other antihypertensive treatment in those patients are not yet available [162].

#### 7.2.3. Salbutamol

Salbutamol is a short-acting β_2_-agonist broadly used as bronchodilator to treat respiratory conditions such as asthma [163]. It has recently been shown how salbutamol stimulation of β_2_-adrenergic receptor mimics sympathetic hyperactivity and leads to NCC-mediated hypertension in mice, but the precise cellular processes triggered by this drug remain to be elucidated. The hypothesis proposed by Poulsen and colleagues is that salbutamol promotes NCC phosphorylation by both stimulating the Kir4.1/5.1-WNK-SPAK/OSR pathway at various levels and inhibiting PP1-mediated NCC dephosphorylation [164]. However, conflicting results have emerged about the role of β_2_-adrenergic receptor in the regulation of blood pressure through WNK-SPAK/OSR1-NCC cascade [165]; therefore, further studies are certainly required for a deeper understanding of salbutamol-induced hypertension.

### 7.3. SGLT2 Inhibitors

SGLT2 inhibitors (SGLT2i, glifozines) are a new, well-tolerated class of glycosuric antidiabetic medications that block the SGLT2 renal glucose transporter, thereby preventing glucose reabsorption and increasing its excretion in urine. Additionally, SGLT2i lowers blood pressure independently of their effect on blood glucose levels, suggesting potential future use as antihypertensive agents [166].

#### 7.3.1. Mechanisms of Action and Blood Pressure Regulation

SGLT2i directly disrupt the function of SGLT2 and NHE3 (sodium hydrogen exchanger 3) in the S1 and S2 segments of the proximal tubule, reducing the reabsorption of sodium, glucose, bicarbonate, and water. However, these effects are balanced by the activation of compensatory pathways for electrolyte and water reabsorption in downstream nephron segments, driven by vasopressin, aldosterone, α-ketoglutarate, carbonic anhydrase, and uromodulin [167]. SGLT2 inhibition has been shown to raise circulating aldosterone levels in some studies, and the increased glucose delivery to the DCT stimulates sodium reabsorption through NCC [168,169]. Additionally, SGLT2i elevates α-ketoglutarate levels in the proximal tubule, which supports sodium and chloride reabsorption via chloride–bicarbonate exchanger pathways and enhances ammoniagenesis to facilitate renal acid excretion [170]. Under normal conditions, the tubular fluid reaching the distal nephron is free of glucose as all filtered glucose is reabsorbed by the sodium–glucose transporters SGLT2 and SGLT1 in the apical membrane of the nephron cells beyond the proximal tubule. However, in individuals with diabetes, the excess filtered glucose often exceeds the capacity for reabsorption, resulting in glucose being delivered to the distal nephron. As for fructose, it is well-established that chronic high fructose intake is linked to the development of hypertension, with several mechanisms proposed to explain this evidence. Since fructose reabsorption in the proximal tubule depends on the amount filtered, high consumption can lead to an increased filtration of fructose, surpassing the re-absorptive capacity and allowing fructose to reach the distal nephron. In this context, the presence of glucose or fructose may act as a calcimimetic, increasing CaSR sensitivity to calcium, enhancing NCC activity, and promoting NaCl reabsorption. This mechanism could contribute to the higher prevalence of hypertension in individuals with diabetes or high fructose consumption. The pathophysiology of BP reduction during treatment with SGLT2i is still not fully understood, though it has attracted increasing attention in recent years. Postulated mechanisms of SGLT2i’s influence on blood pressure include osmotic diuresis, natriuresis, and modulation of the sympathetic nervous system [171].

#### 7.3.2. Gliflozines Influence on NCC Levels and Activation

NCC activity was also shown to be implicated in SGLT2i effects, but, to date, results are not univocal. In obese diabetic mice, ipragliflozin, one of the available SGLT2i, lowered PKC activity in DCT cells and reduced KLHL3 phosphorylation and NCC levels [172]. In another setting, diabetic OLETF rats treated with empagliflozin did not show a significant change in NCC expression in the kidney compared to untreated diabetic rats, while the treatment with lixisenatide caused an increase in the NCC [173]. Similarly, in Dahl salt-sensitive rats on a high-sodium diet, SGLT2i administration did not alter NCC expression (mRNA and protein), while lowering BP [174]. On the contrary, in normotensive and spontaneously hypertensive rats, chronic administration of SGLT2 inhibitors affected apical renal transporters responsible for tubular sodium reabsorption under normal and high BP conditions. The authors found that treatment with empagliflozin inhibits proximal tubule NHE3 activity, augments natriuresis, and increases diuretic and natriuretic responses to a saline challenge in both hypertensive and normotensive rats. They also showed that just in normotensive animals, empagliflozin upregulates NCC function via transcriptional and post-translational mechanisms and lowers BP only in hypertensive but not in normotensive rats [175]. In line with this finding, other authors, in cell lines and animal models showed that SGLT2i, through the stimulus of the Ca^2+^-sensing receptor in the distal convoluted tubule and consequent glycosuria, caused increased activity of NCC. The authors also demonstrated a similar increase in pNCC in UEVs from healthy subjects after treatment with fructose or gliflozins [169,176].

Globally, studies on the effect of SGLT2i on NCC are, at present, not conclusive, and little data are available in humans. Future research should investigate the effect of this class of drugs on different populations and settings, analyzing the different specific effect of the various SGLT2i also in combination with concomitant therapy (e.g., thiazides diuretics).

## 8. NCC in Extracellular Vesicles in Health and Hypertensive Diseases

NCC protein abundance (both NCC and pNCC) is normally assessed by antibody-based methods in tissue samples (kidney biopsies or tissue specimens) usually derived from animals. In humans, however, the availability of kidney tissue samples is hampered by the invasive procedure; therefore, the NCC has recently been investigated in UEVs. EVs originating from urinary tract cells contain molecules derived from glomerular, tubular, and bladder cells [177]. Those EVs released from renal tubules carry protein channels from different segments involved in Na^+^ and water reabsorption under hormonal control [7]. Consequently, analyzing UEVs presents a non-invasive, innovative method of investigating the physiological and pathophysiological regulation of NCC in humans [7,178]. Interestingly, NCC can be detected not only in its protein form in UEVs; the mRNA transcript encoding for NCC can be also evaluated, thus enlarging the potential for deciphering further NCC pathophysiological mechanisms [178]. We recently demonstrated that NCC mRNA is detectable in UEVs, and its expression can provide useful information [144]. The study investigated the modulation of NCC expression under various conditions, including saline infusion, anti-aldosterone treatment, and adrenal surgery. With regards to NCC protein in UEVs, the discovery of the NCC in low-density membrane fractions from the urine of normal rats initially suggested that urine analysis could be valuable in clinical settings [179]. NCC levels in UEVs were found to be significantly increased in individuals with FHHt compared to controls, highlighting the NCC’s key role in FHHt pathophysiology [180]. Treatment with calcineurin inhibitors also elevated NCC and pNCC levels in human UEVs [181], with thiazide diuretics effectively lowering blood pressure in these patients [182]. In Gitelman syndrome, the NCC is either absent or reduced in UEVs, mirroring findings in the patients’ renal tissue [183]. Moreover, NCC levels in UEVs can potentially distinguish between salt-sensitive and salt-resistant hypertensive patients [184].

In addition, in UEVs derived from healthy individuals, the abundance of NCC or pNCC did not differ between high and low Na intake [185]. In accordance with this finding, in urinary exosomes from healthy individuals, it was demonstrated that the NCC and prostasin (a serine protease activating ENaC) followed a circadian pattern largely parallel to that observed for ADH and AQP2, suggesting that both systems are strictly associated with the water-retention rather than the Na^+^ retention (aldosterone, cortisol) pathway [186]. Interestingly, the Stowasser group analyzed the responses to alterations in K^+^ intake in healthy individuals by giving to participants 1 week of a high Na^+^ and high K^+^ diet and 1 week of a high Na^+^ and low K^+^ diet. UEVs were isolated from the subjects before and after each dietary phase, and the abundances of NCC and pNCC were analyzed [187]. Their main results were that the abundance and phosphorylation of NCC in the UEVs of healthy adults on a high-Na^+^ diet was reduced in response to supplementary K^+^ intake. This reduction of NCC confirms the hypothesis of a “renal-K^+^ switch” in humans, providing further support to the assumption that dietary K^+^ interventions are a valuable strategy to lower BP. A similar dietary intervention study was published very recently regarding the effects of a dietary approach to stopping hypertension (DASH diet) in hypertensive human subjects on UEVs proteins [188]. The DASH diet combines the antihypertensive effect of a low-sodium and high-potassium diet. The potassium component of the diet acts as a switch in the DCT to reduce Na^+^ reabsorption. Using mass spectrometry and immunoblotting, the authors detected an increase in total NCC abundance and a decrease in aquaporin-2 in UEVs, together with a significant increase in the pNCC-to-total NCC ratio and a decrease in the AQP2 from day 5 to day 11. The results of this work support the view that the human kidney’s response (in terms of adjustment of the tubular cells and their function) to diet changes, namely, from high to low salt and low to high potassium over a period of 11 days, may be assessed non-invasively by UEV protein abundance variations. A seminal study on a small cohort showed that NCC protein levels in UEVs were higher in patients with PA than in those with essential hypertension (EH). As expected, the patients with PA were characterized by a different urinary ion excretion, with a much lower sodium-to-potassium ratio in patient with PA compared to patients with EH. This finding supports the hypothesis that the NCC could serve as a marker for aldosteronism [189], even if the specific mechanism underlying the NCC modulation has not yet been investigated. Further studies have shown that in hypertensive patients, modulation of the RAAS via a high- or low-sodium diet led to parallel changes in exosomal NCC content [190]. Additionally, the administration of the exogenous mineralocorticoid fludrocortisone resulted in a rapid increase in both NCC and pNCC levels, possibly due to potassium regulation as potassium levels were inversely related to NCC and pNCC [191]. This finding is in agreement with other studies conducted on mice, showing that K^+^ supplementation lessened the extent of NCC abundance upregulation caused by aldosterone infusion [192,193]. This suggests that increased plasma K^+^ levels from KCl supplements during testing might offset the NCC stimulation induced by mineralocorticoids. Recently, the impact of NaCl loading and volume expansion on the UEV NCC profile, as well as the potential role of intravenous NaCl loading alone on the NCC in patients with PA, was assessed. Although there was a notable decrease in plasma aldosterone levels in these patients, no changes were observed in UEV NCC and pNCC after normalization. This indicates that aldosterone may not be a primary regulator of NCC abundance and phosphorylation. On the contrary, the NCC and pNCC in UEVs were decreased after K^+^ supplementation, further emphasizing that extracellular fluid K^+^ concentration is a strong regulator of the NCC [194]. Furthermore, in another recent study, it was found that pNCC and NCC levels were more abundant in patients with PA with unilateral adrenal disease (compared to those with bilateral disease) during adrenal venous sampling. This suggests that UEVs NCC protein could be a potential biomarker for distinguishing PA subtypes, further highlighting its diagnostic value [195].

## 9. Discussion, Conclusions, and Future Directions

### 9.1. General Considerations

The NCC is a key player in blood pressure regulation, and genetic or acquired modifications in NCC activity are prominently implicated in the pathophysiology of arterial hypertension development. Gitelman and Bartter syndromes prove that a genetic impairment altering NCC structure/activation implies an impaired ECF and blood pressure modulation. In recent years, the possible role of the NCC was also investigated in EH and secondary hypertension, particularly in the field of salt-sensitive hypertension and PA. In PA, the increase in sodium retention is imputable principally to ENaC activation, but an influence on NCC activity was also documented. The NCC is modulated by salt intake in both healthy and hypertensive subjects. However, the effective contribution of NCC dysregulation in salt sensitivity is still debated. The few available studies on hypertension secondary to cortisol excess in Cushing syndrome indicate a role for NCC activation in the water and sodium retention characterizing the disease. Alongside its role in water sodium retention, the NCC also seems to be related to hypertension through its interaction with the sympathetic nervous system (mediated by norepinephrine, especially in the context of salt-sensitive hypertension), i.e., with angiotensin II and ADH (both activating the NCC).

### 9.2. NCC in EVs

Most studies on the NCC are performed in vitro or are limited to animal models in vivo due to the difficulty of evaluating the *NCC* gene expression and protein function at the glomerular level of the kidney tissue, where the NCC is mainly located. Thus, UEVs are now considered a valid alternative to renal biopsy for investigating glomerular molecules. The few human studies relating to UEVs analysis, however, are not always consistent with the animal studies, and some discrepancies are evident when analyzing different molecules (for instance, protein and related mRNA). When the NCC was analyzed in relation to salt sensitivity and oral sodium load in animals, a relation was evident between sodium excess and the NCC. In human UEVs, the same relation was not confirmed. Among the possible hypotheses explaining this discrepancy, the option of a waste pathway mechanism should be considered: the increase in NCC abundance in UEVs could either mirror an increase in protein concentration and function or, alternatively, could be due to an increase in EVs shedding to eliminate functionally inactive proteins. In PA, the available studies were globally consistent with an increase in NCC abundance in patients, hypothesizing that the consequent increase in Na^+^ transport could contribute to the volume expansion that characterizes patients with PA. By contrast, in the only study investigating mRNA abundance in the UEVs of patients with PA, there were no differences in NCC expression between patients with EH and those with PA, nor between mRNA levels among the different PA subtypes. UEVs NCC mRNA levels were instead increased after medical or surgical therapy. A possible reason for the differences among studies on UEVs NCC protein and mRNA is the existence of a different modulation of translation and transcription in patients with PA, with a hypothetical inhibition of translation mirroring a feedback mechanism in conditions of high water and sodium retention and different mechanisms enhancing the transcription. Alongside the intrinsic methodological complexity of isolating and analyzing UEVs mRNA, we believe that transcriptomic analysis of UEVs, particularly in the context of understanding mineralocorticoid-related hypertension, could be crucial for advancing our knowledge of both the physiology and pathophysiology of mineralocorticoid hypertension.

### 9.3. Perspectives in Hypertension Modulation

Studies on the NCC underline the relevance of potassium intake in hypertension. Alongside the well-known recommendation for reducing dietary sodium, some of the latest hypertension guidelines cite the possibility of increasing the oral potassium content to aid blood pressure control [196,197]. Potassium interventions have been shown to lower BP [198,199], especially in patients with high sodium intake [196]. It is interesting to highlight how patients with a high sodium intake are characterized by activating renal ion channels to maintain the sodium balance. The effects of potassium on NCC activity are influenced by a range of potassium-mediated changes in cellular signaling, collectively driving what is known as the “renal-K^+^ switch”. The potassium switch suggests that low intake increases NCC activity, whereas a high potassium intake reduces the activation of the NCC. Thus, while aldosterone stimulates ENaC to enhance distal Na^+^ reabsorption and K^+^ excretion, ECF K^+^ concentration directly regulates NCC levels and its phosphorylation. The modulation of the NCC by potassium concentration partially explains the positive effect of potassium supplementation on reducing blood pressure. This mechanism could present new opportunities for pharmaceutical or dietary interventions in both health and disease. In the interest of better controlling blood pressure worldwide [1], actions influencing NCC activity could represent an important therapeutic chance. As a lifestyle modification, increasing dietary potassium or specific potassium supplementation can globally reduce NCC activation. Nevertheless, further studies are required to confirm the positive effect of K^+^ supplementation on BP, and caution should be taken in establishing intake thresholds. A study in mice, in fact, reported that chronic high-K^+^ diet intake promoted a BP increase through an ENaC-mediated mechanisms independently of changes in Na^+^ intake [200].

Moreover, drugs influencing NCC activity already play—or could play in the future—an important role. In recent years, in cellular and animal models, several compounds potentially able to lower blood pressure by interfering with the phosphorylation pathways involved in NCC regulation were studied [201]. The possible sites of pharmacological intervention include the inhibition of WNK kinases [202,203], the inhibition of SPAK/OSR1 [204,205], and the inhibition of the interaction between WNK kinases and SPAK/OSR1 [206]. However, to date, even if the results on blood pressure are interesting for some molecules, the lack of selectivity among the isoforms of target pathways has hindered the advancement of drug discovery studies. Given the relevance of the NCC in blood pressure control, and the efficacy of the inhibition of the NCC by thiazide diuretics in lowering blood pressure, future studies are needed to identify other possible mechanism of inhibiting NCC activation and to determine which specific hypertensive populations could have a better response to thiazide diuretics. As well as patients with salt-sensitive hypertension, specific studies should evaluate the efficacy of thiazide diuretics in patients with primary aldosteronism or other forms of endocrine hypertension (i.e., Cushing syndrome) and hypertension induced by certain drugs, such as calcineurin inhibitors. Finally, the action of SGLT2i on the NCC deserves our full attention, and further studied are urgently needed as these drugs are acquiring a growing importance in the management of patients with heart disease and/or hypertension.

## Data Availability

No new data were created or analyzed in this study. Data sharing is not applicable to this article.

## References

[1] Global Report on Hypertension: The Race Against a Silent Killer. https://www.who.int/publications/i/item/9789240081062.

[2] Mancia G., Kreutz R., Brunström M., Burnier M., Grassi G., Januszewicz A., Muiesan M.L., Tsioufis K., Agabiti-Rosei E., Algharably E.A.E. (2023). 2023 ESH Guidelines for the Management of Arterial Hypertension The Task Force for the Management of Arterial Hypertension of the European Society of Hypertension: Endorsed by the International Society of Hypertension (ISH) and the European Renal Association (ERA). J. Hypertens..

[3] Suzumoto Y., Zucaro L., Iervolino A., Capasso G. (2023). Kidney and Blood Pressure Regulation-Latest Evidence for Molecular Mechanisms. Clin. Kidney J..

[4] Wadei H.M., Textor S.C. (2012). The Role of the Kidney in Regulating Arterial Blood Pressure. Nat. Rev. Nephrol..

[5] Stockand J.D. (2010). Vasopressin Regulation of Renal Sodium Excretion. Kidney Int..

[6] Salih M., Fenton R.A., Zietse R., Hoorn E.J. (2016). Urinary Extracellular Vesicles as Markers to Assess Kidney Sodium Transport. Curr. Opin. Nephrol. Hypertens..

[7] Wu A., Wolley M.J., Fenton R.A., Stowasser M. (2022). Using Human Urinary Extracellular Vesicles to Study Physiological and Pathophysiological States and Regulation of the Sodium Chloride Cotransporter. Front. Endocrinol..

[8] Friso S., Castagna A., Mango G., Olivieri O., Pizzolo F. (2023). Urinary Extracellular Vesicles Carry Valuable Hints through mRNA for the Understanding of Endocrine Hypertension. Front. Endocrinol..

[9] Gamba G. (2005). Molecular Physiology and Pathophysiology of Electroneutral Cation-Chloride Cotransporters. Physiol. Rev..

[10] Gamba G., Saltzberg S.N., Lombardi M., Miyanoshita A., Lytton J., Hediger M.A., Brenner B.M., Hebert S.C. (1993). Primary Structure and Functional Expression of a cDNA Encoding the Thiazide-Sensitive, Electroneutral Sodium-Chloride Cotransporter. Proc. Natl. Acad. Sci. USA.

[11] Arroyo J.P., Kahle K.T., Gamba G. (2013). The SLC12 Family of Electroneutral Cation-Coupled Chloride Cotransporters. Mol. Asp. Med..

[12] Gamba G. (2023). Thirty Years of the NaCl Cotransporter: From Cloning to Physiology and Structure. Am. J. Physiol. Renal Physiol..

[13] Hoorn E.J., Gritter M., Cuevas C.A., Fenton R.A. (2020). Regulation of the Renal NaCl Cotransporter and Its Role in Potassium Homeostasis. Physiol. Rev..

[14] Franken G.A.C., Adella A., Bindels R.J.M., de Baaij J.H.F. (2021). Mechanisms Coupling Sodium and Magnesium Reabsorption in the Distal Convoluted Tubule of the Kidney. Acta Physiol..

[15] Maeoka Y., McCormick J.A. (2020). NaCl Cotransporter Activity and Mg^2+^ Handling by the Distal Convoluted Tubule. Am. J. Physiol. Renal Physiol..

[16] Chávez-Canales M., García J.A., Gamba G. (2023). Regulation of the WNK4-SPAK-NCC Pathway by the Calcium-Sensing Receptor. Curr. Opin. Nephrol. Hypertens..

[17] de Jong J.C., Willems P.H.G.M., Mooren F.J.M., van den Heuvel L.P.W.J., Knoers N.V.A.M., Bindels R.J.M. (2003). The Structural Unit of the Thiazide-Sensitive NaCl Cotransporter Is a Homodimer. J. Biol. Chem..

[18] Nan J., Yuan Y., Yang X., Shan Z., Liu H., Wei F., Zhang W., Zhang Y. (2022). Cryo-EM Structure of the Human Sodium-Chloride Cotransporter NCC. Sci. Adv..

[19] Fan M., Zhang J., Lee C.-L., Zhang J., Feng L. (2023). Structure and Thiazide Inhibition Mechanism of the Human Na-Cl Cotransporter. Nature.

[20] Delpire E., Guo J. (2020). Cryo-EM Structures of DrNKCC1 and hKCC1: A New Milestone in the Physiology of Cation-Chloride Cotransporters. Am. J. Physiol. Cell Physiol..

[21] Lomize M.A., Pogozheva I.D., Joo H., Mosberg H.I., Lomize A.L. (2012). OPM Database and PPM Web Server: Resources for Positioning of Proteins in Membranes. Nucleic Acids Res..

[22] Richardson C., Rafiqi F.H., Karlsson H.K.R., Moleleki N., Vandewalle A., Campbell D.G., Morrice N.A., Alessi D.R. (2008). Activation of the Thiazide-Sensitive Na^+^-Cl^−^ Cotransporter by the WNK-Regulated Kinases SPAK and OSR1. J. Cell Sci..

[23] Zhao Y., Schubert H., Blakely A., Forbush B., Smith M.D., Rinehart J., Cao E. (2024). Structural Bases for Na^+^-Cl^−^ Cotransporter Inhibition by Thiazide Diuretic Drugs and Activation by Kinases. Nat. Commun..

[24] Pacheco-Alvarez D., Cristóbal P.S., Meade P., Moreno E., Vazquez N., Muñoz E., Díaz A., Juárez M.E., Giménez I., Gamba G. (2006). The Na^+^:Cl^−^ Cotransporter Is Activated and Phosphorylated at the Amino-Terminal Domain upon Intracellular Chloride Depletion. J. Biol. Chem..

[25] Mercier-Zuber A., O’Shaughnessy K.M. (2011). Role of SPAK and OSR1 Signalling in the Regulation of NaCl Cotransporters. Curr. Opin. Nephrol. Hypertens..

[26] Rosenbaek L.L., Kortenoeven M.L.A., Aroankins T.S., Fenton R.A. (2014). Phosphorylation Decreases Ubiquitylation of the Thiazide-Sensitive Cotransporter NCC and Subsequent Clathrin-Mediated Endocytosis. J. Biol. Chem..

[27] Rosenbaek L.L., Rizzo F., MacAulay N., Staub O., Fenton R.A. (2017). Functional Assessment of Sodium Chloride Cotransporter NCC Mutants in Polarized Mammalian Epithelial Cells. Am. J. Physiol. Renal Physiol..

[28] Furusho T., Uchida S., Sohara E. (2020). The WNK Signaling Pathway and Salt-Sensitive Hypertension. Hypertens. Res..

[29] Thomson M.N., Cuevas C.A., Bewarder T.M., Dittmayer C., Miller L.N., Si J., Cornelius R.J., Su X.-T., Yang C.-L., McCormick J.A. (2020). WNK Bodies Cluster WNK4 and SPAK/OSR1 to Promote NCC Activation in Hypokalemia. Am. J. Physiol. Renal Physiol..

[30] Rojas-Vega L., Gamba G. (2016). Mini-Review: Regulation of the Renal NaCl Cotransporter by Hormones. Am. J. Physiol. Renal Physiol..

[31] Bazúa-Valenti S., Rojas-Vega L., Castañeda-Bueno M., Barrera-Chimal J., Bautista R., Cervantes-Pérez L.G., Vázquez N., Plata C., Murillo-de-Ozores A.R., González-Mariscal L. (2018). The Calcium-Sensing Receptor Increases Activity of the Renal NCC through the WNK4-SPAK Pathway. J. Am. Soc. Nephrol..

[32] Rosenbaek L.L., Rizzo F., Wu Q., Rojas-Vega L., Gamba G., MacAulay N., Staub O., Fenton R.A. (2017). The Thiazide Sensitive Sodium Chloride Co-Transporter NCC Is Modulated by Site-Specific Ubiquitylation. Sci. Rep..

[33] Rosenbaek L.L., Petrillo F., van Bemmelen M.X., Staub O., Murali S.K., Fenton R.A. (2022). The E3 Ubiquitin-Protein Ligase Nedd4-2 Regulates the Sodium Chloride Cotransporter NCC but Is Not Required for a Potassium-Induced Reduction of NCC Expression. Front. Physiol..

[34] Ohta A., Schumacher F.-R., Mehellou Y., Johnson C., Knebel A., Macartney T.J., Wood N.T., Alessi D.R., Kurz T. (2013). The CUL3–KLHL3 E3 Ligase Complex Mutated in Gordon’s Hypertension Syndrome Interacts with and Ubiquitylates WNK Isoforms: Disease-Causing Mutations in KLHL3 and WNK4 Disrupt Interaction. Biochem. J..

[35] Picard N., Trompf K., Yang C.-L., Miller R.L., Carrel M., Loffing-Cueni D., Fenton R.A., Ellison D.H., Loffing J. (2014). Protein Phosphatase 1 Inhibitor-1 Deficiency Reduces Phosphorylation of Renal NaCl Cotransporter and Causes Arterial Hypotension. J. Am. Soc. Nephrol..

[36] Glover M., Mercier Zuber A., Figg N., O’Shaughnessy K.M. (2010). The Activity of the Thiazide-Sensitive Na(+)-Cl(−) Cotransporter Is Regulated by Protein Phosphatase PP4. Can. J. Physiol. Pharmacol..

[37] Shoda W., Nomura N., Ando F., Mori Y., Mori T., Sohara E., Rai T., Uchida S. (2017). Calcineurin Inhibitors Block Sodium-Chloride Cotransporter Dephosphorylation in Response to High Potassium Intake. Kidney Int..

[38] Ishizawa K., Wang Q., Li J., Yamazaki O., Tamura Y., Fujigaki Y., Uchida S., Lifton R.P., Shibata S. (2019). Calcineurin Dephosphorylates Kelch-like 3, Reversing Phosphorylation by Angiotensin II and Regulating Renal Electrolyte Handling. Proc. Natl. Acad. Sci. USA.

[39] Penton D., Moser S., Wengi A., Czogalla J., Rosenbaek L.L., Rigendinger F., Faresse N., Martins J.R., Fenton R.A., Loffing-Cueni D. (2019). Protein Phosphatase 1 Inhibitor-1 Mediates the cAMP-Dependent Stimulation of the Renal NaCl Cotransporter. J. Am. Soc. Nephrol..

[40] Fanestil D.D. (1992). Steroid Regulation of Thiazide-Sensitive Transport. Semin. Nephrol..

[41] Chen Z., Vaughn D.A., Fanestil D.D. (1994). Influence of Gender on Renal Thiazide Diuretic Receptor Density and Response. J. Am. Soc. Nephrol..

[42] Chen Z.F., Vaughn D.A., Beaumont K., Fanestil D.D. (1990). Effects of Diuretic Treatment and of Dietary Sodium on Renal Binding of 3H-Metolazone. J. Am. Soc. Nephrol..

[43] Shirley D.G., Skinner J., Walter S.J. (1987). The Influence of Dietary Potassium on the Renal Tubular Effect of Hydrochlorothiazide in the Rat. Br. J. Pharmacol..

[44] Tiwari S., Riazi S., Ecelbarger C.A. (2007). Insulin’s Impact on Renal Sodium Transport and Blood Pressure in Health, Obesity, and Diabetes. Am. J. Physiol. Renal Physiol..

[45] Chávez-Canales M., Arroyo J.P., Ko B., Vázquez N., Bautista R., Castañeda-Bueno M., Bobadilla N.A., Hoover R.S., Gamba G. (2013). Insulin Increases the Functional Activity of the Renal NaCl Cotransporter. J. Hypertens..

[46] Komers R., Rogers S., Oyama T.T., Xu B., Yang C.-L., Mccormick J., Ellison D.H. (2012). Enhanced Phosphorylation of Na-Cl Cotransporter in Experimental Metabolic Syndrome—Role of Insulin. Clin. Sci..

[47] Sohara E., Rai T., Yang S.-S., Ohta A., Naito S., Chiga M., Nomura N., Lin S.-H., Vandewalle A., Ohta E. (2011). Acute Insulin Stimulation Induces Phosphorylation of the Na-Cl Cotransporter in Cultured Distal mpkDCT Cells and Mouse Kidney. PLoS ONE.

[48] Nishida H., Sohara E., Nomura N., Chiga M., Alessi D.R., Rai T., Sasaki S., Uchida S. (2012). Phosphatidylinositol 3-Kinase/Akt Signaling Pathway Activates the WNK-OSR1/SPAK-NCC Phosphorylation Cascade in Hyperinsulinemic Db/Db Mice. Hypertension.

[49] Yoshizaki Y., Mori Y., Tsuzaki Y., Mori T., Nomura N., Wakabayashi M., Takahashi D., Zeniya M., Kikuchi E., Araki Y. (2015). Impaired Degradation of WNK by Akt and PKA Phosphorylation of KLHL3. Biochem. Biophys. Res. Commun..

[50] Kortenoeven M.L.A., Pedersen N.B., Rosenbaek L.L., Fenton R.A. (2015). Vasopressin Regulation of Sodium Transport in the Distal Nephron and Collecting Duct. Am. J. Physiol. Renal Physiol..

[51] Saritas T., Borschewski A., McCormick J.A., Paliege A., Dathe C., Uchida S., Terker A., Himmerkus N., Bleich M., Demaretz S. (2013). SPAK Differentially Mediates Vasopressin Effects on Sodium Cotransporters. J. Am. Soc. Nephrol..

[52] Rieg T., Tang T., Uchida S., Hammond H.K., Fenton R.A., Vallon V. (2013). Adenylyl Cyclase 6 Enhances NKCC2 Expression and Mediates Vasopressin-Induced Phosphorylation of NKCC2 and NCC. Am. J. Pathol..

[53] Castañeda-Bueno M., Arroyo J.P., Zhang J., Puthumana J., Yarborough O., Shibata S., Rojas-Vega L., Gamba G., Rinehart J., Lifton R.P. (2017). Phosphorylation by PKC and PKA Regulate the Kinase Activity and Downstream Signaling of WNK4. Proc. Natl. Acad. Sci. USA.

[54] Carbajal-Contreras H., Murillo-de-Ozores A.R., Magaña-Avila G., Marquez-Salinas A., Bourqui L., Tellez-Sutterlin M., Bahena-Lopez J.P., Cortes-Arroyo E., Behn-Eschenburg S.G., Lopez-Saavedra A. (2024). Arginine Vasopressin Regulates the Renal Na^+^-Cl^−^ and Na^+^-K^+^-Cl^−^ Cotransporters through with-No-Lysine Kinase 4 and Inhibitor 1 Phosphorylation. Am. J. Physiol. Renal Physiol..

[55] Cheng L., Wu Q., Kortenoeven M.L.A., Pisitkun T., Fenton R.A. (2015). A Systems Level Analysis of Vasopressin-Mediated Signaling Networks in Kidney Distal Convoluted Tubule Cells. Sci. Rep..

[56] Brooks H.L., Allred A.J., Beutler K.T., Coffman T.M., Knepper M.A. (2002). Targeted Proteomic Profiling of Renal Na(+) Transporter and Channel Abundances in Angiotensin II Type 1a Receptor Knockout Mice. Hypertension.

[57] van der Lubbe N., Zietse R., Hoorn E.J. (2013). Effects of Angiotensin II on Kinase-Mediated Sodium and Potassium Transport in the Distal Nephron. Curr. Opin. Nephrol. Hypertens..

[58] San-Cristobal P., Pacheco-Alvarez D., Richardson C., Ring A.M., Vazquez N., Rafiqi F.H., Chari D., Kahle K.T., Leng Q., Bobadilla N.A. (2009). Angiotensin II Signaling Increases Activity of the Renal Na-Cl Cotransporter through a WNK4-SPAK-Dependent Pathway. Proc. Natl. Acad. Sci. USA.

[59] Ko B., Mistry A., Hanson L., Mallick R., Hoover R.S. (2015). Mechanisms of Angiotensin II Stimulation of NCC Are Time-Dependent in mDCT15 Cells. Am. J. Physiol. Renal Physiol..

[60] Shibata S., Arroyo J.P., Castañeda-Bueno M., Puthumana J., Zhang J., Uchida S., Stone K.L., Lam T.T., Lifton R.P. (2014). Angiotensin II Signaling via Protein Kinase C Phosphorylates Kelch-like 3, Preventing WNK4 Degradation. Proc. Natl. Acad. Sci. USA.

[61] Veiras L.C., Han J., Ralph D.L., McDonough A.A. (2016). Potassium Supplementation Prevents Sodium Chloride Cotransporter Stimulation During Angiotensin II Hypertension. Hypertension.

[62] Ivy J.R., Jones N.K., Costello H.M., Mansley M.K., Peltz T.S., Flatman P.W., Bailey M.A. (2019). Glucocorticoid Receptor Activation Stimulates the Sodium-Chloride Cotransporter and Influences the Diurnal Rhythm of Its Phosphorylation. Am. J. Physiol. Renal Physiol..

[63] Canonica J., Frateschi S., Boscardin E., Ebering A., Sergi C., Jäger Y., Peyrollaz T., Mérillat A.-M., Maillard M., Klusonova P. (2019). Lack of Renal Tubular Glucocorticoid Receptor Decreases the Thiazide-Sensitive Na^+^/Cl^−^ Cotransporter NCC and Transiently Affects Sodium Handling. Front. Physiol..

[64] Ivy J.R., Oosthuyzen W., Peltz T.S., Howarth A.R., Hunter R.W., Dhaun N., Al-Dujaili E.A.S., Webb D.J., Dear J.W., Flatman P.W. (2016). Glucocorticoids Induce Nondipping Blood Pressure by Activating the Thiazide-Sensitive Cotransporter. Hypertension.

[65] Lou Y., Zhang F., Luo Y., Wang L., Huang S., Jin F. (2016). Serum and Glucocorticoid Regulated Kinase 1 in Sodium Homeostasis. Int. J. Mol. Sci..

[66] Rashmi P., Colussi G., Ng M., Wu X., Kidwai A., Pearce D. (2017). Glucocorticoid-Induced Leucine Zipper Protein Regulates Sodium and Potassium Balance in the Distal Nephron. Kidney Int..

[67] Richards J., Ko B., All S., Cheng K.-Y., Hoover R.S., Gumz M.L. (2014). A Role for the Circadian Clock Protein Per1 in the Regulation of the NaCl Co-Transporter (NCC) and the with-No-Lysine Kinase (WNK) Cascade in Mouse Distal Convoluted Tubule Cells. J. Biol. Chem..

[68] Rojas-Vega L., Reyes-Castro L.A., Ramírez V., Bautista-Pérez R., Rafael C., Castañeda-Bueno M., Meade P., de Los Heros P., Arroyo-Garza I., Bernard V. (2015). Ovarian Hormones and Prolactin Increase Renal NaCl Cotransporter Phosphorylation. Am. J. Physiol. Renal Physiol..

[69] Veiras L.C., Girardi A.C.C., Curry J., Pei L., Ralph D.L., Tran A., Castelo-Branco R.C., Pastor-Soler N., Arranz C.T., Yu A.S.L. (2017). Sexual Dimorphic Pattern of Renal Transporters and Electrolyte Homeostasis. J. Am. Soc. Nephrol..

[70] Verlander J.W., Tran T.M., Zhang L., Kaplan M.R., Hebert S.C. (1998). Estradiol Enhances Thiazide-Sensitive NaCl Cotransporter Density in the Apical Plasma Membrane of the Distal Convoluted Tubule in Ovariectomized Rats. J. Clin. Investig..

[71] Tahaei E., Coleman R., Saritas T., Ellison D.H., Welling P.A. (2020). Distal Convoluted Tubule Sexual Dimorphism Revealed by Advanced 3D Imaging. Am. J. Physiol. Renal Physiol..

[72] Preston R.A., Norris P.M., Alonso A.B., Ni P., Hanes V., Karara A.H. (2007). Randomized, Placebo-Controlled Trial of the Effects of Drospirenone-Estradiol on Blood Pressure and Potassium Balance in Hypertensive Postmenopausal Women Receiving Hydrochlorothiazide. Menopause.

[73] Posadzy-Malaczynska A., Rajpold K., Woznicka-Leskiewicz L., Marcinkowska J. (2015). Hemodynamic and Metabolic Effects of Estrogen plus Progestin Therapy in Hypertensive Postmenopausal Women Treated with an ACE-Inhibitor or a Diuretic. Clin. Res. Cardiol..

[74] Kim G.H., Masilamani S., Turner R., Mitchell C., Wade J.B., Knepper M.A. (1998). The Thiazide-Sensitive Na-Cl Cotransporter Is an Aldosterone-Induced Protein. Proc. Natl. Acad. Sci. USA.

[75] van der Lubbe N., Lim C.H., Meima M.E., van Veghel R., Rosenbaek L.L., Mutig K., Danser A.H.J., Fenton R.A., Zietse R., Hoorn E.J. (2012). Aldosterone Does Not Require Angiotensin II to Activate NCC through a WNK4-SPAK-Dependent Pathway. Pflugers Arch..

[76] Terker A.S., Yarbrough B., Ferdaus M.Z., Lazelle R.A., Erspamer K.J., Meermeier N.P., Park H.J., McCormick J.A., Yang C.-L., Ellison D.H. (2016). Direct and Indirect Mineralocorticoid Effects Determine Distal Salt Transport. J. Am. Soc. Nephrol..

[77] Kristensen M., Fenton R.A., Poulsen S.B. (2022). Dissecting the Effects of Aldosterone and Hypokalemia on the Epithelial Na^+^ Channel and the NaCl Cotransporter. Front. Physiol..

[78] Cheng L., Poulsen S.B., Wu Q., Esteva-Font C., Olesen E.T.B., Peng L., Olde B., Leeb-Lundberg L.M.F., Pisitkun T., Rieg T. (2019). Rapid Aldosterone-Mediated Signaling in the DCT Increases Activity of the Thiazide-Sensitive NaCl Cotransporter. J. Am. Soc. Nephrol..

[79] Chiga M., Rai T., Yang S.-S., Ohta A., Takizawa T., Sasaki S., Uchida S. (2008). Dietary Salt Regulates the Phosphorylation of OSR1/SPAK Kinases and the Sodium Chloride Cotransporter through Aldosterone. Kidney Int..

[80] Arroyo J.P., Lagnaz D., Ronzaud C., Vázquez N., Ko B.S., Moddes L., Ruffieux-Daidié D., Hausel P., Koesters R., Yang B. (2011). Nedd4-2 Modulates Renal Na^+^-Cl^−^ Cotransporter via the Aldosterone-SGK1-Nedd4-2 Pathway. J. Am. Soc. Nephrol..

[81] Terker A.S., Zhang C., McCormick J.A., Lazelle R.A., Zhang C., Meermeier N.P., Siler D.A., Park H.J., Fu Y., Cohen D.M. (2015). Potassium Modulates Electrolyte Balance and Blood Pressure through Effects on Distal Cell Voltage and Chloride. Cell Metab..

[82] Sorensen M.V., Grossmann S., Roesinger M., Gresko N., Todkar A.P., Barmettler G., Ziegler U., Odermatt A., Loffing-Cueni D., Loffing J. (2013). Rapid Dephosphorylation of the Renal Sodium Chloride Cotransporter in Response to Oral Potassium Intake in Mice. Kidney Int..

[83] Rioux A.V., Nsimba-Batomene T.R., Slimani S., Bergeron N.A., Gravel M.A., Schreiber S.V., Fiola M.J., Haydock L., Garneau A.P., Isenring P. (2024). Navigating the Multifaceted Intricacies of the Na^+^-Cl^−^ Cotransporter, a Highly Regulated Key Effector in the Control of Hydromineral Homeostasis. Physiol. Rev..

[84] Blanchard A., Bockenhauer D., Bolignano D., Calò L.A., Cosyns E., Devuyst O., Ellison D.H., Karet Frankl F.E., Knoers N.V.A.M., Konrad M. (2017). Gitelman Syndrome: Consensus and Guidance from a Kidney Disease: Improving Global Outcomes (KDIGO) Controversies Conference. Kidney Int..

[85] Parmar M.S., Muppidi V., Bashir K. (2024). Gitelman Syndrome. StatPearls.

[86] Loffing J., Vallon V., Loffing-Cueni D., Aregger F., Richter K., Pietri L., Bloch-Faure M., Hoenderop J.G.J., Shull G.E., Meneton P. (2004). Altered Renal Distal Tubule Structure and Renal Na(+) and Ca(2+) Handling in a Mouse Model for Gitelman’s Syndrome. J. Am. Soc. Nephrol..

[87] Mrad F.C.C., Soares S.B.M., de Menezes Silva L.A.W., dos Anjos Menezes P.V., Simões-e-Silva A.C. (2021). Bartter’s Syndrome: Clinical Findings, Genetic Causes and Therapeutic Approach. World J. Pediatr..

[88] Laghmani K., Beck B.B., Yang S.-S., Seaayfan E., Wenzel A., Reusch B., Vitzthum H., Priem D., Demaretz S., Bergmann K. (2016). Polyhydramnios, Transient Antenatal Bartter’s Syndrome, and MAGED2 Mutations. N. Engl. J. Med..

[89] Teulon J., Planelles G., Sepúlveda F.V., Andrini O., Lourdel S., Paulais M. (2018). Renal Chloride Channels in Relation to Sodium Chloride Transport. Comprehensive Physiology.

[90] Piala A.T., Moon T.M., Akella R., He H., Cobb M.H., Goldsmith E.J. (2014). Chloride Sensing by WNK1 Involves Inhibition of Autophosphorylation. Sci. Signal..

[91] Johnston J.G., Wingo C.S. (2022). Potassium Homeostasis and WNK Kinases in the Regulation of the Sodium-Chloride Cotransporter: Hyperaldosteronism and Its Metabolic Consequences. Kidney360.

[92] Lo J., Forst A.-L., Warth R., Zdebik A.A. (2022). EAST/SeSAME Syndrome and Beyond: The Spectrum of Kir4.1- and Kir5.1-Associated Channelopathies. Front. Physiol..

[93] Palazzo V., Raglianti V., Landini S., Cirillo L., Errichiello C., Buti E., Artuso R., Tiberi L., Vergani D., Dirupo E. (2022). Clinical and Genetic Characterization of Patients with Bartter and Gitelman Syndrome. Int. J. Mol. Sci..

[94] Manas F., Singh S. (2024). Pseudohypoaldosteronism Type II or Gordon Syndrome: A Rare Syndrome of Hyperkalemia and Hypertension With Normal Renal Function. Cureus.

[95] Mabillard H., Sayer J.A. (2019). The Molecular Genetics of Gordon Syndrome. Genes.

[96] Sohara E., Uchida S. (2016). Kelch-like 3/Cullin 3 Ubiquitin Ligase Complex and WNK Signaling in Salt-Sensitive Hypertension and Electrolyte Disorder. Nephrol. Dial. Transplant..

[97] Alessi D.R., Zhang J., Khanna A., Hochdörfer T., Shang Y., Kahle K.T. (2014). The WNK-SPAK/OSR1 Pathway: Master Regulator of Cation-Chloride Cotransporters. Sci. Signal..

[98] Vidal-Petiot E., Elvira-Matelot E., Mutig K., Soukaseum C., Baudrie V., Wu S., Cheval L., Huc E., Cambillau M., Bachmann S. (2013). WNK1-Related Familial Hyperkalemic Hypertension Results from an Increased Expression of L-WNK1 Specifically in the Distal Nephron. Proc. Natl. Acad. Sci. USA.

[99] Kim S.M., Eisner C., Faulhaber-Walter R., Mizel D., Wall S.M., Briggs J.P., Schnermann J. (2008). Salt Sensitivity of Blood Pressure in NKCC1-Deficient Mice. Am. J. Physiol. Renal Physiol..

[100] Liang L., Shimosawa T. (2023). Molecular Mechanisms of Na-Cl Cotransporter in Relation to Hypertension in Chronic Kidney Disease. Int. J. Mol. Sci..

[101] Welling P.A., Little R., Al-Qusairi L., Delpire E., Ellison D.H., Fenton R.A., Grimm P.R. (2024). Potassium-Switch Signaling Pathway Dictates Acute Blood Pressure Response to Dietary Potassium. Hypertension.

[102] Terker A.S., Zhang C., Erspamer K.J., Gamba G., Yang C.-L., Ellison D.H. (2016). Unique Chloride-Sensing Properties of WNK4 Permit the Distal Nephron to Modulate Potassium Homeostasis. Kidney Int..

[103] Canonica J., Sergi C., Maillard M., Klusonova P., Odermatt A., Koesters R., Loffing-Cueni D., Loffing J., Rossier B., Frateschi S. (2016). Adult Nephron-Specific MR-Deficient Mice Develop a Severe Renal PHA-1 Phenotype. Pflugers Arch..

[104] Czogalla J., Vohra T., Penton D., Kirschmann M., Craigie E., Loffing J. (2016). The Mineralocorticoid Receptor (MR) Regulates ENaC but Not NCC in Mice with Random MR Deletion. Pflugers Arch..

[105] Hundemer G.L. (2019). Primary Aldosteronism: Cardiovascular Outcomes Pre- and Post-Treatment. Curr. Cardiol. Rep..

[106] Thout S.R., Santos J.A., McKenzie B., Trieu K., Johnson C., McLean R., Arcand J., Campbell N.R.C., Webster J. (2019). The Science of Salt: Updating the Evidence on Global Estimates of Salt Intake. J. Clin. Hypertens..

[107] Hunter R.W., Dhaun N., Bailey M.A. (2022). The Impact of Excessive Salt Intake on Human Health. Nat. Rev. Nephrol..

[108] Adolf C., Görge V., Heinrich D.A., Hoster E., Schneider H., Handgriff L., Künzel H., Sturm L., Beuschlein F., Reincke M. (2021). Altered Taste Perception for Sodium Chloride in Patients With Primary Aldosteronism: A Prospective Cohort Study. Hypertension.

[109] Mutchler S.M., Hasan M., Murphy C.P., Baty C.J., Boyd-Shiwarski C., Kirabo A., Kleyman T.R. (2024). Dietary Sodium Alters Aldosterone’s Effect on Renal Sodium Transporter Expression and Distal Convoluted Tubule Remodelling. J. Physiol..

[110] Zhou L., Jiang Y., Zhang C., Su T., Jiang L., Zhou W., Zhong X., Wu L., Wang W. (2023). Effects of a Low-Sodium Diet in Patients with Idiopathic Hyperaldosteronism: A Randomized Controlled Trial. Front. Endocrinol..

[111] Pilic L., Pedlar C.R., Mavrommatis Y. (2016). Salt-Sensitive Hypertension: Mechanisms and Effects of Dietary and Other Lifestyle Factors. Nutr. Rev..

[112] Lerman L.O., Kurtz T.W., Touyz R.M., Ellison D.H., Chade A.R., Crowley S.D., Mattson D.L., Mullins J.J., Osborn J., Eirin A. (2019). Animal Models of Hypertension: A Scientific Statement From the American Heart Association. Hypertension.

[113] Kim K., Nist K.M., Puleo F., McKenna J., Wainford R.D. (2024). Sex Differences in Dietary Sodium Evoked NCC Regulation and Blood Pressure in Male and Female Sprague-Dawley, Dahl Salt-Resistant, and Dahl Salt-Sensitive Rats. Am. J. Physiol. Renal Physiol..

[114] Rodriguez-Iturbe B., Vaziri N.D. (2007). Salt-Sensitive Hypertension--Update on Novel Findings. Nephrol. Dial. Transplant..

[115] Kopp U.C. (2015). Role of Renal Sensory Nerves in Physiological and Pathophysiological Conditions. Am. J. Physiol. Regul. Integr. Comp. Physiol..

[116] Sonalker P.A., Tofovic S.P., Bastacky S.I., Jackson E.K. (2008). Chronic Noradrenaline Increases Renal Expression of NHE-3, NBC-1, BSC-1 and Aquaporin-2. Clin. Exp. Pharmacol. Physiol..

[117] Mu S., Shimosawa T., Ogura S., Wang H., Uetake Y., Kawakami-Mori F., Marumo T., Yatomi Y., Geller D.S., Tanaka H. (2011). Epigenetic Modulation of the Renal β-Adrenergic-WNK4 Pathway in Salt-Sensitive Hypertension. Nat. Med..

[118] Terker A.S., Yang C.-L., McCormick J.A., Meermeier N.P., Rogers S.L., Grossmann S., Trompf K., Delpire E., Loffing J., Ellison D.H. (2014). Sympathetic Stimulation of Thiazide-Sensitive Sodium Chloride Cotransport in the Generation of Salt-Sensitive Hypertension. Hypertension.

[119] Wainford R.D., Carmichael C.Y., Pascale C.L., Kuwabara J.T. (2015). Gαi2-Protein-Mediated Signal Transduction: Central Nervous System Molecular Mechanism Countering the Development of Sodium-Dependent Hypertension. Hypertension.

[120] Walsh K.R., Kuwabara J.T., Shim J.W., Wainford R.D. (2016). Norepinephrine-Evoked Salt-Sensitive Hypertension Requires Impaired Renal Sodium Chloride Cotransporter Activity in Sprague-Dawley Rats. Am. J. Physiol. Regul. Integr. Comp. Physiol..

[121] Frame A.A., Puleo F., Kim K., Walsh K.R., Faudoa E., Hoover R.S., Wainford R.D. (2019). Sympathetic Regulation of NCC in Norepinephrine-Evoked Salt-Sensitive Hypertension in Sprague-Dawley Rats. Am. J. Physiol. Renal Physiol..

[122] Puleo F., Kim K., Frame A.A., Walsh K.R., Ferdaus M.Z., Moreira J.D., Comsti E., Faudoa E., Nist K.M., Abkin E. (2020). Sympathetic Regulation of the NCC (Sodium Chloride Cotransporter) in Dahl Salt-Sensitive Hypertension. Hypertension.

[123] Doutova E.A., Moss N.G. (1996). Age-Related Changes in Calcitonin Gene-Related Peptide and Substance P in Renal Afferent Nerve Soma in the Rat. Association with Afferent Renal Nerve Activity. Brain Res. Dev. Brain Res..

[124] Frame A.A., Nist K.M., Kim K., Puleo F., Moreira J.D., Swaldi H., McKenna J., Wainford R.D. (2024). Integrated Renal and Sympathetic Mechanisms Underlying the Development of Sex- and Age-Dependent Hypertension and the Salt Sensitivity of Blood Pressure. Geroscience.

[125] Cicala M.V., Mantero F. (2010). Hypertension in Cushing’s Syndrome: From Pathogenesis to Treatment. Neuroendocrinology.

[126] Feelders R.A., Pulgar S.J., Kempel A., Pereira A.M. (2012). The Burden of Cushing’s Disease: Clinical and Health-Related Quality of Life Aspects. Eur. J. Endocrinol..

[127] Hunter R.W., Ivy J.R., Bailey M.A. (2014). Glucocorticoids and Renal Na^+^ Transport: Implications for Hypertension and Salt Sensitivity. J. Physiol..

[128] Frindt G., Palmer L.G. (2012). Regulation of Epithelial Na^+^ Channels by Adrenal Steroids: Mineralocorticoid and Glucocorticoid Effects. Am. J. Physiol. Renal Physiol..

[129] Velázquez H., Bartiss A., Bernstein P., Ellison D.H. (1996). Adrenal Steroids Stimulate Thiazide-Sensitive NaCl Transport by Rat Renal Distal Tubules. Am. J. Physiol..

[130] Salih M., Bovée D.M., van der Lubbe N., Danser A.H.J., Zietse R., Feelders R.A., Hoorn E.J. (2018). Increased Urinary Extracellular Vesicle Sodium Transporters in Cushing Syndrome With Hypertension. J. Clin. Endocrinol. Metab..

[131] Tranquilli A.L., Dekker G., Magee L., Roberts J., Sibai B.M., Steyn W., Zeeman G.G., Brown M.A. (2014). The Classification, Diagnosis and Management of the Hypertensive Disorders of Pregnancy: A Revised Statement from the ISSHP. Pregnancy Hypertens..

[132] Brown M.A., Wang J., Whitworth J.A. (1997). The Renin-Angiotensin-Aldosterone System in Pre-Eclampsia. Clin. Exp. Hypertens..

[133] Hu C.-C., Katerelos M., Choy S.-W., Crossthwaite A., Walker S.P., Pell G., Lee M., Cook N., Mount P.F., Paizis K. (2018). Pre-Eclampsia Is Associated with Altered Expression of the Renal Sodium Transporters NKCC2, NCC and ENaC in Urinary Extracellular Vesicles. PLoS ONE.

[134] Wang P., Zhu G., Wu Q., Shen L., Liu D., Wang Z., Wang W., Ren Z., Jia Y., Liu M. (2023). Renal CD81 Interacts with Sodium Potassium 2 Chloride Cotransporter and Sodium Chloride Cotransporter in Rats with Lipopolysaccharide-Induced Preeclampsia. FASEB J..

[135] Brown A., Meor Azlan N.F., Wu Z., Zhang J. (2021). WNK-SPAK/OSR1-NCC Kinase Signaling Pathway as a Novel Target for the Treatment of Salt-Sensitive Hypertension. Acta Pharmacol. Sin..

[136] Ernst M.E., Moser M. (2009). Use of Diuretics in Patients with Hypertension. N. Engl. J. Med..

[137] Ernst M.E., Fravel M.A. (2022). Thiazide and the Thiazide-Like Diuretics: Review of Hydrochlorothiazide, Chlorthalidone, and Indapamide. Am. J. Hypertens..

[138] Roush G.C., Ernst M.E., Kostis J.B., Tandon S., Sica D.A. (2015). Head-to-Head Comparisons of Hydrochlorothiazide with Indapamide and Chlorthalidone: Antihypertensive and Metabolic Effects. Hypertension.

[139] Palmer B.F., Naderi A.S.A. (2007). Metabolic Complications Associated with Use of Thiazide Diuretics. J. Am. Soc. Hypertens..

[140] Shao S.-C., Lai C.-C., Chen Y.-H., Lai E.C.-C., Hung M.-J., Chi C.-C. (2022). Associations of Thiazide Use with Skin Cancers: A Systematic Review and Meta-Analysis. BMC Med..

[141] Kreutz R., Algharably E.A.H., Douros A. (2019). Reviewing the Effects of Thiazide and Thiazide-like Diuretics as Photosensitizing Drugs on the Risk of Skin Cancer. J. Hypertens..

[142] Abdallah J.G., Schrier R.W., Edelstein C., Jennings S.D., Wyse B., Ellison D.H. (2001). Loop Diuretic Infusion Increases Thiazide-Sensitive Na(+)/Cl(−)-Cotransporter Abundance: Role of Aldosterone. J. Am. Soc. Nephrol..

[143] Nielsen J., Kwon T.-H., Masilamani S., Beutler K., Hager H., Nielsen S., Knepper M.A. (2002). Sodium Transporter Abundance Profiling in Kidney: Effect of Spironolactone. Am. J. Physiol. Renal Physiol..

[144] Pizzolo F., Bertolone L., Castagna A., Morandini F., Sartori G., De Santis D., Tiberti N., Brazzarola P., Salvagno G., Friso S. (2022). Urinary Extracellular Vesicle mRNA Analysis of Sodium Chloride Cotransporter in Hypertensive Patients under Different Conditions. J. Hum. Hypertens..

[145] Kolkhof P., Jaisser F., Kim S.-Y., Filippatos G., Nowack C., Pitt B. (2017). Steroidal and Novel Non-Steroidal Mineralocorticoid Receptor Antagonists in Heart Failure and Cardiorenal Diseases: Comparison at Bench and Bedside. Handb. Exp. Pharmacol..

[146] Susa K., Sohara E., Isobe K., Chiga M., Rai T., Sasaki S., Uchida S. (2012). WNK-OSR1/SPAK-NCC Signal Cascade Has Circadian Rhythm Dependent on Aldosterone. Biochem. Biophys. Res. Commun..

[147] Pathare G., Anderegg M., Albano G., Lang F., Fuster D.G. (2018). Elevated FGF23 Levels in Mice Lacking the Thiazide-Sensitive NaCl Cotransporter (NCC). Sci. Rep..

[148] Rao V.S., Planavsky N., Hanberg J.S., Ahmad T., Brisco-Bacik M.A., Wilson F.P., Jacoby D., Chen M., Tang W.H.W., Cherney D.Z.I. (2017). Compensatory Distal Reabsorption Drives Diuretic Resistance in Human Heart Failure. J. Am. Soc. Nephrol..

[149] Loon N.R., Wilcox C.S., Unwin R.J. (1989). Mechanism of Impaired Natriuretic Response to Furosemide during Prolonged Therapy. Kidney Int..

[150] Wilcox C.S., Testani J.M., Pitt B. (2020). Pathophysiology of Diuretic Resistance and Its Implications for the Management of Chronic Heart Failure. Hypertension.

[151] Reilly R.F., Ellison D.H. (2000). Mammalian Distal Tubule: Physiology, Pathophysiology, and Molecular Anatomy. Physiol. Rev..

[152] Obermüller N., Bernstein P., Velázquez H., Reilly R., Moser D., Ellison D.H., Bachmann S. (1995). Expression of the Thiazide-Sensitive Na-Cl Cotransporter in Rat and Human Kidney. Am. J. Physiol..

[153] van Angelen A.A., van der Kemp A.W., Hoenderop J.G., Bindels R.J. (2012). Increased Expression of Renal TRPM6 Compensates for Mg(2+) Wasting during Furosemide Treatment. Clin. Kidney J..

[154] Safarini O.A., Keshavamurthy C., Patel P. (2024). Calcineurin Inhibitors. StatPearls.

[155] Ho S., Clipstone N., Timmermann L., Northrop J., Graef I., Fiorentino D., Nourse J., Crabtree G.R. (1996). The Mechanism of Action of Cyclosporin A and FK506. Clin. Immunol. Immunopathol..

[156] Farouk S.S., Rein J.L. (2020). The Many Faces of Calcineurin Inhibitor Toxicity-What the FK?. Adv. Chronic Kidney Dis..

[157] Hošková L., Málek I., Kopkan L., Kautzner J. (2017). Pathophysiological Mechanisms of Calcineurin Inhibitor-Induced Nephrotoxicity and Arterial Hypertension. Physiol. Res..

[158] Hoorn E.J., Walsh S.B., McCormick J.A., Fürstenberg A., Yang C.-L., Roeschel T., Paliege A., Howie A.J., Conley J., Bachmann S. (2011). The Calcineurin Inhibitor Tacrolimus Activates the Renal Sodium Chloride Cotransporter to Cause Hypertension. Nat. Med..

[159] Duan X.-P., Zhang C.-B., Wang W.-H., Lin D.-H. (2024). Role of Calcineurin in Regulating Renal Potassium (K^+^) Excretion: Mechanisms of Calcineurin Inhibitor-Induced Hyperkalemia. Acta Physiol..

[160] Shoda W., Nomura N., Ando F., Tagashira H., Iwamoto T., Ohta A., Isobe K., Mori T., Susa K., Sohara E. (2020). Sodium-Calcium Exchanger 1 Is the Key Molecule for Urinary Potassium Excretion against Acute Hyperkalemia. PLoS ONE.

[161] Gao Z.-X., Zhou R., Li M.-Y., Li S.-T., Mao Z.-H., Shu T.-T., Liu D.-W., Liu Z.-S., Wu P. (2023). Activation of Kir4.1/Kir5.1 Contributes to the Cyclosporin A-Induced Stimulation of the Renal NaCl Cotransporter and Hyperkalemic Hypertension. Acta Physiol..

[162] Hoorn E.J., Walsh S.B., McCormick J.A., Zietse R., Unwin R.J., Ellison D.H. (2012). Pathogenesis of Calcineurin Inhibitor-Induced Hypertension. J. Nephrol..

[163] Marques L., Vale N. (2022). Salbutamol in the Management of Asthma: A Review. Int. J. Mol. Sci..

[164] Poulsen S.B., Cheng L., Penton D., Kortenoeven M.L.A., Matchkov V.V., Loffing J., Little R., Murali S.K., Fenton R.A. (2021). Activation of the Kidney Sodium Chloride Cotransporter by the Β2-Adrenergic Receptor Agonist Salbutamol Increases Blood Pressure. Kidney Int..

[165] Kamiar A., Yousefi K., Dunkley J.C., Webster K.A., Shehadeh L.A. (2021). Β2-Adrenergic Receptor Agonism as a Therapeutic Strategy for Kidney Disease. Am. J. Physiol. Regul. Integr. Comp. Physiol..

[166] Zhang Q., Zhou S., Liu L. (2023). Efficacy and Safety Evaluation of SGLT2i on Blood Pressure Control in Patients with Type 2 Diabetes and Hypertension: A New Meta-Analysis. Diabetol. Metab. Syndr..

[167] Packer M., Wilcox C.S., Testani J.M. (2023). Critical Analysis of the Effects of SGLT2 Inhibitors on Renal Tubular Sodium, Water and Chloride Homeostasis and Their Role in Influencing Heart Failure Outcomes. Circulation.

[168] Zanchi A., Burnier M., Muller M.-E., Ghajarzadeh-Wurzner A., Maillard M., Loncle N., Milani B., Dufour N., Bonny O., Pruijm M. (2020). Acute and Chronic Effects of SGLT2 Inhibitor Empagliflozin on Renal Oxygenation and Blood Pressure Control in Nondiabetic Normotensive Subjects: A Randomized, Placebo-Controlled Trial. J. Am. Heart Assoc..

[169] Bahena-Lopez J.P., Rojas-Vega L., Chávez-Canales M., Bazua-Valenti S., Bautista-Pérez R., Lee J.-H., Madero M., Vazquez-Manjarrez N., Alquisiras-Burgos I., Hernandez-Cruz A. (2023). Glucose/Fructose Delivery to the Distal Nephron Activates the Sodium-Chloride Cotransporter via the Calcium-Sensing Receptor. J. Am. Soc. Nephrol..

[170] Onishi A., Fu Y., Patel R., Darshi M., Crespo-Masip M., Huang W., Song P., Freeman B., Kim Y.C., Soleimani M. (2020). A Role for Tubular Na^+^/H^+^ Exchanger NHE3 in the Natriuretic Effect of the SGLT2 Inhibitor Empagliflozin. Am. J. Physiol. Renal Physiol..

[171] Ahwin P., Martinez D. (2024). The Relationship between SGLT2 and Systemic Blood Pressure Regulation. Hypertens. Res..

[172] Ishizawa K., Wang Q., Li J., Xu N., Nemoto Y., Morimoto C., Fujii W., Tamura Y., Fujigaki Y., Tsukamoto K. (2019). Inhibition of Sodium Glucose Cotransporter 2 Attenuates the Dysregulation of Kelch-Like 3 and NaCl Cotransporter in Obese Diabetic Mice. J. Am. Soc. Nephrol..

[173] Chung S., Kim S., Son M., Kim M., Koh E.S., Shin S.J., Ko S.-H., Kim H.-S. (2019). Empagliflozin Contributes to Polyuria via Regulation of Sodium Transporters and Water Channels in Diabetic Rat Kidneys. Front. Physiol..

[174] Kravtsova O., Bohovyk R., Levchenko V., Palygin O., Klemens C.A., Rieg T., Staruschenko A. (2022). SGLT2 Inhibition Effect on Salt-Induced Hypertension, RAAS, and Na^+^ Transport in Dahl SS Rats. Am. J. Physiol. Renal Physiol..

[175] Castro P.C., Santos-Rios T.M., Martins F.L., Crajoinas R.O., Caetano M.V., Lessa L.M.A., Luchi W.M., McCormick J.A., Girardi A.C.C. (2024). Renal Upregulation of NCC Counteracts Empagliflozin-Mediated NHE3 Inhibition in Normotensive but Not in Hypertensive Male Rat. Am. J. Physiol. Cell Physiol..

[176] Palmer B.F., Clegg D.J. (2024). SGLT2 Inhibition and Kidney Potassium Homeostasis. Clin. J. Am. Soc. Nephrol..

[177] Spanu S., van Roeyen C.R.C., Denecke B., Floege J., Mühlfeld A.S. (2014). Urinary Exosomes: A Novel Means to Non-Invasively Assess Changes in Renal Gene and Protein Expression. PLoS ONE.

[178] Barros E.R., Carvajal C.A. (2017). Urinary Exosomes and Their Cargo: Potential Biomarkers for Mineralocorticoid Arterial Hypertension?. Front. Endocrinol..

[179] McKEE J.A., Kumar S., Ecelbarger C.A., Fernández-Llama P., Terris J., Knepper M.A. (2000). Detection of Na(+) Transporter Proteins in Urine. J. Am. Soc. Nephrol..

[180] Mayan H., Attar-Herzberg D., Shaharabany M., Holtzman E.J., Farfel Z. (2008). Increased Urinary Na-Cl Cotransporter Protein in Familial Hyperkalaemia and Hypertension. Nephrol. Dial. Transplant..

[181] Esteva-Font C., Guillén-Gómez E., Diaz J.M., Guirado L., Facundo C., Ars E., Ballarin J.A., Fernández-Llama P. (2014). Renal Sodium Transporters Are Increased in Urinary Exosomes of Cyclosporine-Treated Kidney Transplant Patients. Am. J. Nephrol..

[182] Tutakhel O.A.Z., Moes A.D., Valdez-Flores M.A., Kortenoeven M.L.A., Vrie M.V.D., Jeleń S., Fenton R.A., Zietse R., Hoenderop J.G.J., Hoorn E.J. (2017). NaCl Cotransporter Abundance in Urinary Vesicles Is Increased by Calcineurin Inhibitors and Predicts Thiazide Sensitivity. PLoS ONE.

[183] Joo K.W., Lee J.W., Jang H.R., Heo N.J., Jeon U.S., Oh Y.K., Lim C.S., Na K.Y., Kim J., Cheong H.I. (2007). Reduced Urinary Excretion of Thiazide-Sensitive Na-Cl Cotransporter in Gitelman Syndrome: Preliminary Data. Am. J. Kidney Dis..

[184] Esteva-Font C., Wang X., Ars E., Guillén-Gómez E., Sans L., González Saavedra I., Torres F., Torra R., Masilamani S., Ballarín J.A. (2010). Are Sodium Transporters in Urinary Exosomes Reliable Markers of Tubular Sodium Reabsorption in Hypertensive Patients?. Nephron Physiol..

[185] Zachar R., Jensen B.L., Svenningsen P. (2019). Dietary Na^+^ Intake in Healthy Humans Changes the Urine Extracellular Vesicle Prostasin Abundance While the Vesicle Excretion Rate, NCC, and ENaC Are Not Altered. Am. J. Physiol. Renal Physiol..

[186] Castagna A., Pizzolo F., Chiecchi L., Morandini F., Channavajjhala S.K., Guarini P., Salvagno G., Olivieri O. (2015). Circadian Exosomal Expression of Renal Thiazide-Sensitive NaCl Cotransporter (NCC) and Prostasin in Healthy Individuals. Proteom. Clin. Appl..

[187] Wu A., Wolley M.J., Mayr H.L., Cheng L., Cowley D., Li B., Campbell K.L., Terker A.S., Ellison D.H., Welling P.A. (2023). Randomized Trial on the Effect of Oral Potassium Chloride Supplementation on the Thiazide-Sensitive Sodium Chloride Cotransporter in Healthy Adults. Kidney Int. Rep..

[188] Bielopolski D., Musante L., Hoorn E.J., Molina H., Barrows D., Carrol T.S., Harding M.A., Upson S., Qureshi A., Weder M.M. (2024). Effect of the DASH Diet on the Sodium-Chloride Cotransporter and Aquaporin-2 in Urinary Extracellular Vesicles. Am. J. Physiol. Renal Physiol..

[189] van der Lubbe N., Jansen P.M., Salih M., Fenton R.A., van den Meiracker A.H., Danser A.H.J., Zietse R., Hoorn E.J. (2012). The Phosphorylated Sodium Chloride Cotransporter in Urinary Exosomes Is Superior to Prostasin as a Marker for Aldosteronism. Hypertension.

[190] Qi Y., Wang X., Rose K.L., MacDonald W.H., Zhang B., Schey K.L., Luther J.M. (2016). Activation of the Endogenous Renin-Angiotensin-Aldosterone System or Aldosterone Administration Increases Urinary Exosomal Sodium Channel Excretion. J. Am. Soc. Nephrol..

[191] Wolley M.J., Wu A., Xu S., Gordon R.D., Fenton R.A., Stowasser M. (2017). In Primary Aldosteronism, Mineralocorticoids Influence Exosomal Sodium-Chloride Cotransporter Abundance. J. Am. Soc. Nephrol..

[192] Xu N., Hirohama D., Ishizawa K., Chang W.X., Shimosawa T., Fujita T., Uchida S., Shibata S. (2017). Hypokalemia and Pendrin Induction by Aldosterone. Hypertension.

[193] Shibata S., Rinehart J., Zhang J., Moeckel G., Castañeda-Bueno M., Stiegler A.L., Boggon T.J., Gamba G., Lifton R.P. (2013). Mineralocorticoid Receptor Phosphorylation Regulates Ligand Binding and Renal Response to Volume Depletion and Hyperkalemia. Cell Metab..

[194] Wu A., Wolley M.J., Matthews A., Cowley D., Welling P.A., Fenton R.A., Stowasser M. (2022). In Primary Aldosteronism Acute Potassium Chloride Supplementation Suppresses Abundance and Phosphorylation of the Sodium-Chloride Cotransporter. Kidney360.

[195] Kong L., Tang X., Kang Y., Dong L., Tong J., Xu J., Gao P., Wang J., Shen W., Zhu L. (2022). The Role of Urinary Extracellular Vesicles Sodium Chloride Cotransporter in Subtyping Primary Aldosteronism. Front. Endocrinol..

[196] Whelton P.K., Carey R.M., Aronow W.S., Casey D.E., Collins K.J., Dennison Himmelfarb C., DePalma S.M., Gidding S., Jamerson K.A., Jones D.W. (2018). 2017 ACC/AHA/AAPA/ABC/ACPM/AGS/APhA/ASH/ASPC/NMA/PCNA Guideline for the Prevention, Detection, Evaluation, and Management of High Blood Pressure in Adults: A Report of the American College of Cardiology/American Heart Association Task Force on Clinical Practice Guidelines. J. Am. Coll. Cardiol..

[197] Rabi D.M., McBrien K.A., Sapir-Pichhadze R., Nakhla M., Ahmed S.B., Dumanski S.M., Butalia S., Leung A.A., Harris K.C., Cloutier L. (2020). Hypertension Canada’s 2020 Comprehensive Guidelines for the Prevention, Diagnosis, Risk Assessment, and Treatment of Hypertension in Adults and Children. Can. J. Cardiol..

[198] Whelton P.K., He J., Cutler J.A., Brancati F.L., Appel L.J., Follmann D., Klag M.J. (1997). Effects of Oral Potassium on Blood Pressure. Meta-Analysis of Randomized Controlled Clinical Trials. JAMA.

[199] Geleijnse J.M., Kok F.J., Grobbee D.E. (2003). Blood Pressure Response to Changes in Sodium and Potassium Intake: A Metaregression Analysis of Randomised Trials. J. Hum. Hypertens..

[200] Little R., Murali S.K., Poulsen S.B., Grimm P.R., Assmus A., Cheng L., Ivy J.R., Hoorn E.J., Matchkov V., Welling P.A. (2023). Dissociation of Sodium-Chloride Cotransporter Expression and Blood Pressure during Chronic High Dietary Potassium Supplementation. JCI Insight.

[201] Meor Azlan N.F., Koeners M.P., Zhang J. (2021). Regulatory Control of the Na-Cl Co-Transporter NCC and Its Therapeutic Potential for Hypertension. Acta Pharm. Sin. B.

[202] Yamada K., Zhang J.-H., Xie X., Reinhardt J., Xie A.Q., LaSala D., Kohls D., Yowe D., Burdick D., Yoshisue H. (2016). Discovery and Characterization of Allosteric WNK Kinase Inhibitors. ACS Chem. Biol..

[203] Yamada K., Levell J., Yoon T., Kohls D., Yowe D., Rigel D.F., Imase H., Yuan J., Yasoshima K., DiPetrillo K. (2017). Optimization of Allosteric With-No-Lysine (WNK) Kinase Inhibitors and Efficacy in Rodent Hypertension Models. J. Med. Chem..

[204] Lin S.-H., Yu I.-S., Jiang S.-T., Lin S.-W., Chu P., Chen A., Sytwu H.-K., Sohara E., Uchida S., Sasaki S. (2011). Impaired Phosphorylation of Na(+)-K(+)-2Cl(−) Cotransporter by Oxidative Stress-Responsive Kinase-1 Deficiency Manifests Hypotension and Bartter-like Syndrome. Proc. Natl. Acad. Sci. USA.

[205] Kikuchi E., Mori T., Zeniya M., Isobe K., Ishigami-Yuasa M., Fujii S., Kagechika H., Ishihara T., Mizushima T., Sasaki S. (2015). Discovery of Novel SPAK Inhibitors That Block WNK Kinase Signaling to Cation Chloride Transporters. J. Am. Soc. Nephrol..

[206] Mori T., Kikuchi E., Watanabe Y., Fujii S., Ishigami-Yuasa M., Kagechika H., Sohara E., Rai T., Sasaki S., Uchida S. (2013). Chemical Library Screening for WNK Signalling Inhibitors Using Fluorescence Correlation Spectroscopy. Biochem. J..

