# Correction: Castagna et al. Sodium Chloride Cotransporter in Hypertension. *Biomedicines* 2024, *12*, 2580

**DOI:** 10.3390/biomedicines14030490

**Published:** 2026-02-24

**Authors:** Annalisa Castagna, Gabriele Mango, Nicola Martinelli, Luigi Marzano, Sara Moruzzi, Simonetta Friso, Francesca Pizzolo

**Affiliations:** 1 Department of Medicine, University of Verona, 37134 Verona, Italy; annalisa.castagna@univr.it (A.C.); gabriele.mango@univr.it (G.M.); nicola.martinelli@univr.it (N.M.); simonetta.friso@univr.it (S.F.); 2 Unit of Internal Medicine B, Department of Medicine, University of Verona School of Medicine, Azienda Ospedaliera Universitaria Integrata Verona, Policlinico “G.B. Rossi”, 37134 Verona, Italy; luigi.marzano@aovr.veneto.it (L.M.); sara.moruzzi@aovr.veneto.it (S.M.)

## Missing Citation

In the original publication [1], Figures 1–5 were created by BioRender, but the proper citation was not included in the figures’ captions.

The figure captions have now been updated as follows:

- **Figure 1.** Sodium reabsorption along the nephron. Most sodium reabsorption occurs in the proximal tubule (pink) via NHE3, which mediates about 70% of the Na^+^ transport, followed by NKCC2 in the thick ascending limb (green), which is responsible for 25% of transport. About 5% of Na^+^ reabsorption occurs in the distal tubule, which includes the distal convoluted tubule (purple) and the collecting duct (magenta). The NCC is located only in the proximal part of the distal convoluted tubule, while ENaC is present in both the distal convoluted tubule and the collecting duct. A representation of the NCC dimeric structure is also illustrated in the image. Created in BioRender. Friso, S. (2026) https://BioRender.com/7ozy4sj (accessed on 8 November 2024).
- **Figure 2.** Three-dimensional structure of the NCC dimer embedded in the apical cell membrane. This schematic representation highlights the functional domains of the transporter: (1) the extracellular domain (green); (2) the transmembrane domain (red); and (3) the cytosolic domain (blue). A close-up of one ion-binding pocket, located within the transmembrane domain, shows three binding sites: one for Na^+^ (magenta); one for Cl^−^ (cyan); and one for polythiazide, a thiazide diuretic (yellow). The representation is based on the structural prediction provided by the Orientations of Proteins in Membranes (OPM) database [21] of the NCC inward-facing conformation obtained by Fan et al. [19] (PDB ID: 8FHO). PyMOL (Schrödinger, LLC) was used to visualize the structure and generate the image. Created in BioRender. Friso, S. (2026) https://BioRender.com/qdks1mm (accessed on 8 November 2024).
- **Figure 3.** Post-translational modifications regulating NCC activity in the DCT. NCC phosphorylation (yellow) is primarily mediated by the WNK-SPAK/OSR1 kinase cascade. WNK1 and WNK4 (with-no-lysine kinases) activate SPAK (STE20/SPS1-related proline/alanine-rich kinases) and OSR1 (oxidative stress-response protein 1), which, in turn, phosphorylate the NCC, increasing its transport activity in the apical membrane. WNK autophosphorylation is stimulated by low Cl^−^ intracellular levels, which result from a decrease in K^+^ intracellular concentration. These changes are, respectively, mediated by basolateral chlorine (light blue) and potassium channels (pink). Cab39 (calcium-binding protein 39) is another regulator of NCC phosphorylation since it is required for SPAK activation. NCC dephosphorylation (red) leads to a reduction in its activity and is carried out by protein phosphatases PP1, PP3, and PP4. PP1 is regulated by protein phosphatase 1 inhibitor-1 (I-1) through the cAMP/PKA pathway. A rise in cAMP levels activates PKA (protein kinase A), which phosphorylates I-1, preventing PP1 from dephosphorylating the NCC. Ubiquitination (purple) is another key regulatory mechanism that affects NCC function. WNK abundance is controlled by an E3 ubiquitin ligase complex composed of KLHL3 (Kelch-like protein 3) and CUL3 (Cullin 3). This complex tags the NCC with ubiquitin (Ub), marking it for proteasomal degradation. KLHL3 acts as an adapter for WNK, and its phosphorylation prevents this binding. PP3 is responsible for KLHL3 activation through dephosphorylation. Additionally, NEDD4-2, another ubiquitin ligase, directly mediates NCC degradation through ubiquitination. Created in BioRender. Friso, S. (2026) https://BioRender.com/ypw9014 (accessed on 8 November 2024).
- **Figure 4.** Physiological stimuli that modulate NCC activity. The key signaling pathways involved in NCC regulation by various hormones are illustrated. Arginine vasopressin (AVP, blue) binds to the V_2_ receptor (V_2_R) on the basolateral membrane, activating adenylate cyclase 6 (AC6), which increases cyclic AMP (cAMP) levels. Elevated cAMP stimulates protein kinase A (PKA), leading to (a) stimulation of WNK-SPAK/OSR1-NCC cascade through WNK kinase phosphorylation; (b) inhibition of protein phosphatase 1 (PP1) by phosphorylating its inhibitor, I-1, maintaining the phosphorylated state of the NCC; and (c) phosphorylation and inactivation of Kelch-like protein 3 (KLHL3), diminishing WNK ubiquitination and consequent degradation. Aldosterone (ALDO, orange) enhances the NCC in both a mineralocorticoid receptor (MR)-dependent and MR-independent manner. Binding to MR, it can stimulate serum- and glucocorticoid-regulated kinase 1 (SGK1), which activates WNKs and simultaneously inhibits NEDD4-2-mediated ubiquitination. On the other hand, aldosterone stimulates ENaC-mediated potassium secretion, eventually inducing hypokalemia. Low extracellular K^+^ levels are sensed by DCT cells through Kir4.1/Kir5.1 K^+^ channels, which, once active, lead to a drop in K^+^ intracellular concentration, stimulating basolateral Cl^-^ transport through ClC-Kb. A decrease in intracellular Cl^−^ levels releases the inhibition of WNK kinases, enabling their autophosphorylation and, thus, stimulating the WNK-SPAK/OSR1-NCC pathway. Cortisol (CORT, red) may exert the same effect as aldosterone, likely through the interaction with the glucocorticoid receptor (GR). Angiotensin II (ANGII, purple) binds to the angiotensin II type 1 receptor (AT1R), activating protein kinase C (PKC), which catalyzes both WNK and KLHL3 phosphorylation. Angiotensin II activates the NCC via AngII receptor type 1 (AT1R), stimulating the WNK4-SPAK kinase cascade and the phosphorylation of KLHL. Angiotensin II can also induce aldosterone and ENaC-mediated hypokalemia, thereby triggering potassium sensing through Kir4.1/Kir5.1 K^+^ channels. Insulin (INS, green) activates the phosphoinositide 3-kinase (PI3K) pathway, leading to the activation of protein kinase B (AKT). AKT stimulates NCC activity through the phosphorylation of both WNK kinases and KLHL3. Created in BioRender. Friso, S. (2026) https://BioRender.com/xp6rgf6 (accessed on 8 November 2024).
- **Figure 5.** Pharmacological modulation of NCC function in the DCT. Inhibitors are shown in red, while enhancers are shown in light blue. Thiazide diuretics directly inhibit NCC, blocking ion translocation, while mineralocorticoid receptor antagonists (MRA) act indirectly, blocking the MR-mediated effect of aldosterone. Loop diuretics (LD) activate the NCC, increasing the amount of sodium delivered to the DCT and the secretion of aldosterone, which increases NCC activity in both an MR-dependent and an MR-independent manner. Calcineurin inhibitors (CNIs) decrease PP3-mediated dephosphorylation of NCC, basolateral K^+^ channel, and KLHL3, thus stimulating NCC activity at various levels. Salbutamol (SALB) could activate the basolateral K^+^ channel through a signaling pathway likely mediated by β_2_-adrenergic receptor. A drop in K^+^ intracellular concentration eventually triggers the WNK-SPAK/OSR1-NCC cascade. SGLT2 inhibitors (SGLT2i, shown in purple) may influence NCC activity, increasing the delivery of glucose and fructose to the DCT and possibly stimulating a signaling pathway involving the calcium-sensing receptor (CaSR) and protein kinase C (PKC). Created in BioRender. Friso, S. (2026) https://BioRender.com/yew9781 (accessed on 8 November 2024).

Citation of Reference 6 was incorrectly added to the captions of the original Figures 1–5 and has been removed. Since Reference 6 is not present in the reference list, only citations to it have been deleted. Citations of References 7–206 have been adjusted to 6–205 to match the reference list, while the last citation [206] remains unchanged and consistent with the list.

The authors state that the scientific conclusions are unaffected. This correction was approved by the Academic Editor. The original publication has also been updated.

## References

[1] Castagna A., Mango G., Martinelli N., Marzano L., Moruzzi S., Friso S., Pizzolo F. (2024). Sodium Chloride Cotransporter in Hypertension. Biomedicines.

